# Identification of Microbial-Based Natural Products as Potential CYP51 Inhibitors for Eumycetoma Treatment: Insights from Molecular Docking, MM-GBSA Calculations, ADMET Analysis, and Molecular Dynamics Simulations

**DOI:** 10.3390/ph18040598

**Published:** 2025-04-20

**Authors:** Tilal Elsaman, Mohamed Khalid Alhaj Awadalla, Malik Suliman Mohamed, Eyman Mohamed Eltayib, Magdi Awadalla Mohamed

**Affiliations:** 1 Department of Pharmaceutical Chemistry, College of Pharmacy, Jouf University, Sakaka 72388, Saudi Arabia; 2 Pharmacy Program, Wad Medani College of Medical Sciences and Technology, Wad Medani 21111, Gezira, Sudan; 3 Department of Pharmaceutics, College of Pharmacy, Jouf University, Sakaka 72388, Saudi Arabia; msmustafa@ju.edu.sa (M.S.M.); emeahmed@ju.edu.sa (E.M.E.)

**Keywords:** eumycetoma, in silico drug discovery, CYP51, homology modeling, natural products

## Abstract

**Background/Objectives:** Eumycetoma, caused by *Madurella mycetomatis*, is a chronic fungal infection with limited treatment options and increasing drug resistance. CYP51, a key enzyme in ergosterol biosynthesis, is a well-established target for azole antifungals. However, existing azole drugs demonstrate limited efficacy in treating eumycetoma. Microbial-based natural products, with their structural diversity and bioactivity, offer a promising source for novel CYP51 inhibitors. This study aimed to identify potential *Madurella mycetomatis* CYP51 inhibitors from microbial natural products using molecular docking, MM-GBSA calculations, ADMET analysis, and molecular dynamics (MD) simulations. **Methods:** Virtual screening was conducted on a library of microbial-based natural products using an in-house homology model of *Madurella mycetomatis* CYP51, with itraconazole as the reference drug. The top compounds from initial docking were refined through Standard and Extra Precision docking. MM-GBSA calculations assessed binding affinities, and ADMET analysis evaluated drug-like properties. Compounds with favorable properties underwent MD simulations. **Results:** The computational investigations identified 34 compounds with better docking scores and binding affinity than itraconazole. Of these, 9 compounds interacted with the heme group and key residues in the active site of *Madurella mycetomatis* CYP51. In silico pharmacokinetic profiling identified 3 compounds as promising candidates, and MD simulations confirmed their potential as CYP51 inhibitors. **Conclusions:** The study highlights microbial-derived natural products, particularly monacyclinone G, H, and I, as promising candidates for *Madurella mycetomatis* CYP51 inhibition, with the potential for treating eumycetoma, requiring further experimental validation.

## 1. Introduction

Eumycetoma, a chronic granulomatous infection caused by the fungal pathogen *Madurella mycetomatis*, is a debilitating condition predominantly affecting individuals in endemic regions. It was officially recognized as a neglected tropical disease (NTD) by the World Health Assembly in 2016 [1,2]. The disease primarily targets the skin, subcutaneous tissues, and sometimes bone—most commonly in the feet, hands, or lower legs. Clinically, it presents as a painless, indurated subcutaneous mass with multiple discharging sinuses and visible grains in the purulent discharge. Over time, the infection can progress, spreading to deeper tissues and causing severe deformities, functional disability, and, if untreated, death [1,2,3,4,5,6,7,8]. The treatment of eumycetoma remains a significant clinical challenge, as commonly used antifungal therapies such as itraconazole, terbinafine, and liposomal amphotericin B often exhibit limited efficacy due to various biological and pharmacological barriers [9,10,11]. These include poor drug penetration into fibrotic tissue, the presence of melanin within fungal grains, and excessive collagen encapsulation around the infection site [7]. Ketoconazole, once widely used, has been withdrawn from many markets due to severe toxicity [12,13]. Surgical intervention is often necessary to manage advanced cases, yet it carries a high risk of recurrence [6]. Compounding these issues are diagnostic delays and inadequate access to medical care in resource-limited endemic areas, which further complicate disease management [14].

Eumycetoma is most common in rural, agricultural communities within tropical and subtropical regions, particularly in the ‘mycetoma belt’ across parts of Africa, Latin America, and Asia [15,16]. While cases have been reported globally, the disease burden is heaviest in underserved areas [1]. Most reported cases of mycetoma have come from Sudan, Mexico, and India, with Sudan standing out as a major hotspot for eumycetoma [16,17,18]. Recent data indicates that the number of eumycetoma cases exceeds 50 per 10,000 inhabitants [19]. The spread of eumycetoma is further driven by factors such as climate change, global travel, increased human-animal-environment interactions, and the resilience of the causative fungal organisms [20,21].

Despite its wide distribution, the true global burden of mycetoma remains uncertain. According to the World Health Organization (WHO), this is largely due to the absence of a standardized case reporting system and a lack of accurate data on incidence and prevalence [15,16]. As a neglected tropical disease, eumycetoma receives minimal attention from the research and pharmaceutical sectors, despite its significant impact on public health. This neglect underscores the urgent need for more effective, affordable, and accessible diagnostic tools as well as new therapeutic strategies [3,5,20].

**Figure 1:**
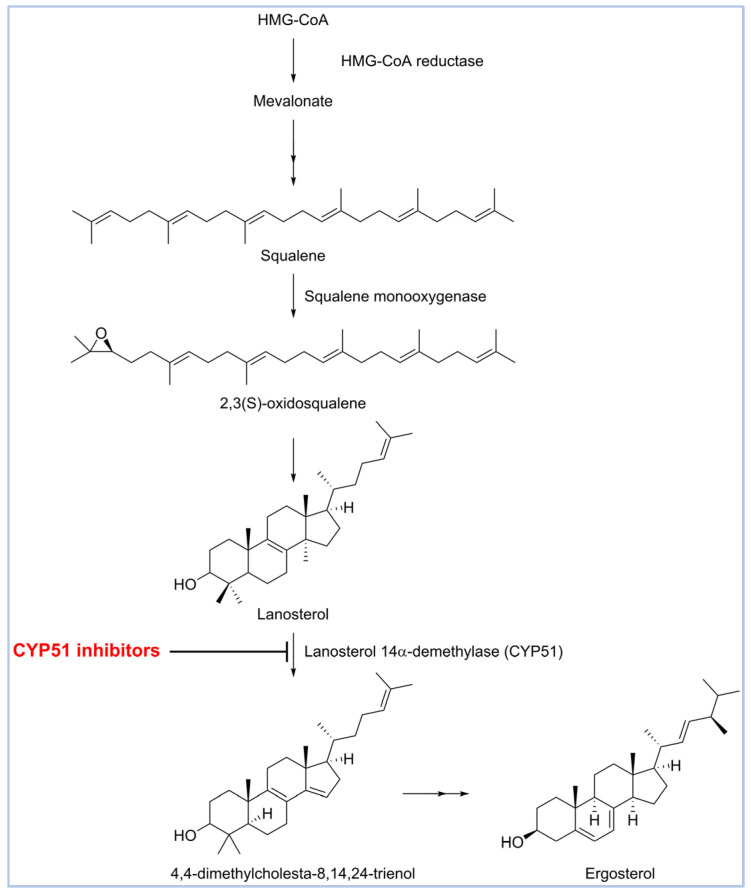
Biosynthesis of ergosterol.

Drug discovery for eumycetoma has been limited by a poor understanding of the disease’s molecular basis and the lack of structural data for key targets such as *Madurella mycetomatis* CYP51 (Cytochrome P450 sterol 14α-demethylase), a crucial enzyme for azole antifungal activity [22]. CYP51 plays a vital role in the ergosterol biosynthesis pathway (Figure 1) and is a well-validated target for antifungal drug discovery [23,24]. Blocking this enzyme prevents the conversion of lanosterol to ergosterol, a critical component of fungal cell membranes [25]. The resulting lack of ergosterol increases the membrane’s permeability, disrupting its integrity and leading to cell lysis and death [26,27].

While azole antifungals are widely used to target CYP51, their limitations in treating eumycetoma necessitate the search for alternative inhibitors with improved potency and safety profiles [11,17]. In a recent unprecedented study, our group employed an integrated computational approach—including homology modeling, virtual screening, free energy calculations, and molecular dynamics simulations—to repurpose FDA-approved drugs as potential treatments [22]. From a library of 2619 compounds, we identified montelukast, vilanterol, and lidoflazine as promising candidates, based on their strong binding affinities, key molecular interactions, and stability in simulations, offering a foundation for future therapeutic development.

**Figure 2:**
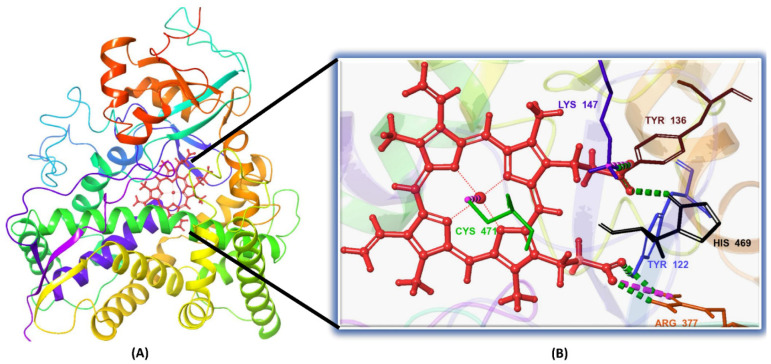
(**A**) The 3D structure of the in-house homology model of *Madurella mycetomatis* CYP51 incorporating the heme cofactor. (**B**) Close-up view of the heme-binding region, showing key interactions. Hydrophobic interactions have been omitted for clarity.

Natural products offer a rich source of novel, potent, and biologically active compounds. They complement synthetic drug approaches by providing leads with unique structures and mechanisms, making them essential in the ongoing search for new therapies—especially in areas of high unmet medical need. Continuing our efforts in drug discovery for eumycetoma, we have expanded the scope to explore microbiologically derived natural products as potential inhibitors of the in-house *Madurella mycetomatis* CYP51 homology model (Figure 2).

## 2. Results and Discussion

Drug discovery is a complex and multidisciplinary process aimed at identifying new therapeutic agents for various diseases [28]. It involves target identification, hit discovery, lead optimization, and preclinical and clinical testing [29]. Traditional drug discovery is often time-consuming and costly, making it particularly challenging for diseases that affect low-income populations, such as neglected tropical diseases (NTDs) [30,31,32]. NTDs, including eumycetoma, receive limited investment despite their significant global burden [2,33,34,35]. To accelerate drug discovery for these diseases, in silico approaches have emerged as powerful tools [36,37,38]. Computational techniques such as virtual screening, molecular docking, molecular dynamics (MD) simulations, and ADMET (Absorption, Distribution, Metabolism, Excretion, and Toxicity) predictions enable researchers to identify and optimize drug candidates efficiently [39,40]. These methods reduce costs and improve the success rate by prioritizing the most promising compounds before laboratory validation, ultimately contributing to the development of effective treatments for NTDs [41,42].

Natural products are renowned for their structural diversity and bioactivity, making them a valuable resource for discovering novel antifungal agents [43]. Given the limited treatment options for eumycetoma, screening microbial-based natural products against *Madurella mycetomatis* druggable targets, such as CYP51, could identify promising drug candidates with enhanced efficacy and selectivity. Although the CYP51 enzyme of *Madurella mycetomatis* was identified in 2016, its 3D structure has yet to be resolved through X-ray crystallography or other experimental methods [44,45]. This limitation has hindered the application of structure-based in silico drug discovery for eumycetoma treatment [46]. To address this gap, we recently constructed a homology model of *Madurella mycetomatis* CYP51 and screened it against a library of FDA-approved drugs to identify potential candidates for repurposing [22]. Building on this effort, we report herein the identification of microbial-based natural products as promising *Madurella mycetomatis* CYP51 inhibitors through an integrated computational approach, paving the way for new therapeutic options for eumycetoma. In this study, the in-house homology model of *Madurella mycetomatis* CYP51 was utilized to screen a microbial-based natural product library [47,48,49] using Schrödinger’s virtual screening workflow [50,51], with itraconazole as a reference drug [52]. The top-ranked, in terms of docking scores, natural products were further evaluated for binding affinity using molecular mechanics-generalized Born surface area (MM-GBSA) calculations [53,54]. Additionally, ADMET studies were conducted to assess the drug-likeness and safety profiles of the promising candidates [55,56]. To gain deeper insights into the stability and dynamics of the ligand-target interactions, MD simulations were performed on the most promising candidates [57,58,59].

### 2.1. Homology Modeling and Binding Site Identification

The primary structure of the *Madurella mycetomatis* CYP51 enzyme, composed of 529 amino acids (available at: https://www.ncbi.nlm.nih.gov/protein/KXX80456, accessed on 1 March 2023), was derived from the first genome sequence of *Madurella mycetomatis*. This strain, identified by the locus tag “MMYC01_202883”, was isolated from a human eumycetoma case in Sudan [44]. A BLASTp search against the Protein Data Bank (PDB) (www.rcsb.org, accessed on 1 March 2023) identified the X-ray crystallography-resolved sterol 14α-demethylase CYP51B from *Aspergillus fumigatus* (PDB ID: 6CR2) [60] as the closest homolog. It showed the highest sequence identity (64.71%), the lowest E-value (0.0), 89% sequence coverage, and similar Max and Total scores (665). A pairwise protein sequence alignment between *Madurella mycetomatis* CYP51 (query) and *Aspergillus fumigatus* CYP51B (subject) was generated using NCBI BLASTp [22]. Based on these results, the *Aspergillus fumigatus* CYP51B structure (PDB ID: 6CR2) was used as a template to construct a homology model of the *Madurella mycetomatis* CYP51 enzyme. The model was built using the YASARA protein modeling software (http://www.yasara.org/general.htm, accessed on 27 December 2024) [61], based on the FASTA-formatted sequence of *Madurella mycetomatis* CYP51 (accession number: KXX80456).

Structural validation of the *Madurella mycetomatis* CYP51 homology model confirmed its reliability. Ramachandran plot analysis via MolProbity (http://molprobity.biochem.duke.edu, accessed on 2 March 2023) showed that 97.1% of residues were in favored regions, with only one outlier, mirroring the quality of the *Aspergillus fumigatus* CYP51B template (PDB: 6CR2). Verify3D (https://www.doe-mbi.ucla.edu, accessed on 5 March 2023) analysis revealed that 85.69% of residues passed the folding reliability threshold, indicating good structural compatibility. Additionally, the model’s QMEAN (https://swissmodel.expasy.org/qmean/, accessed on 9 March 2023) z-score fell within the range typical of high-quality native proteins, further supporting its accuracy. These evaluations validated the model’s suitability for future computational screening studies targeting eumycetoma.

**Figure 3:**
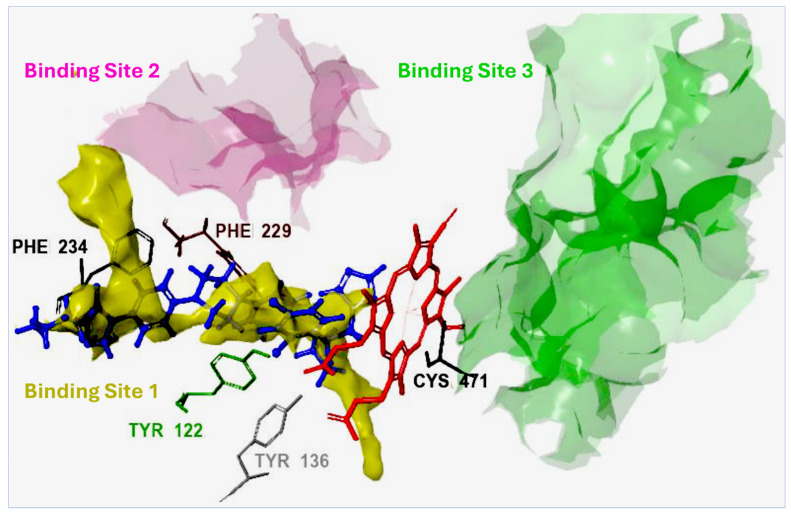
Sitemap prediction of potential binding sites in the *Madurella mycetomatis* CYP51 homology model. Three binding sites were identified with the following Dscores: Site 1 (1.307), Site 2 (1.077), and Site 3 (1.009). Site 1, with the highest Dscore, was selected for grid generation and molecular docking. The reference compound itraconazole (shown in blue) docked favorably within Site 1, forming electrostatic interactions with key amino acid residues (hydrophobic interactions are omitted for clarity). The heme group, depicted in red, is coordinated with the sulfide group of Cys471 through metal coordination.

With the *Madurella mycetomatis* CYP51 homology model constructed, the next step was to identify its potential binding site. For this purpose, the SiteMap tool (Schrödinger Release 2023-1: SiteMap, Schrödinger, LLC, New York, NY, USA, 2023) was employed to predict and score potential binding pockets within the model. A key output of SiteMap is the Dscore, which estimates the druggability of each predicted site—that is, the likelihood that the site can effectively bind drug-like molecules. Higher Dscore values indicate more favorable, druggable pockets and help prioritize targets for further investigation [62]. In our study, the binding site with the highest Dscore (Figure 3) was selected for receptor grid generation and used in subsequent molecular docking studies.

**Figure 4:**
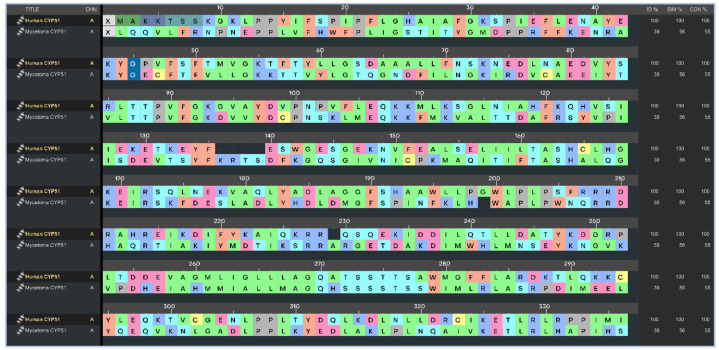
Multiple sequence alignment highlighting identity, similarity, and conserved regions between human CYP51 (PDB ID: 6UEZ) and the homology-modeled CYP51 of *Madurella mycetomatis*.

**Figure 5:**
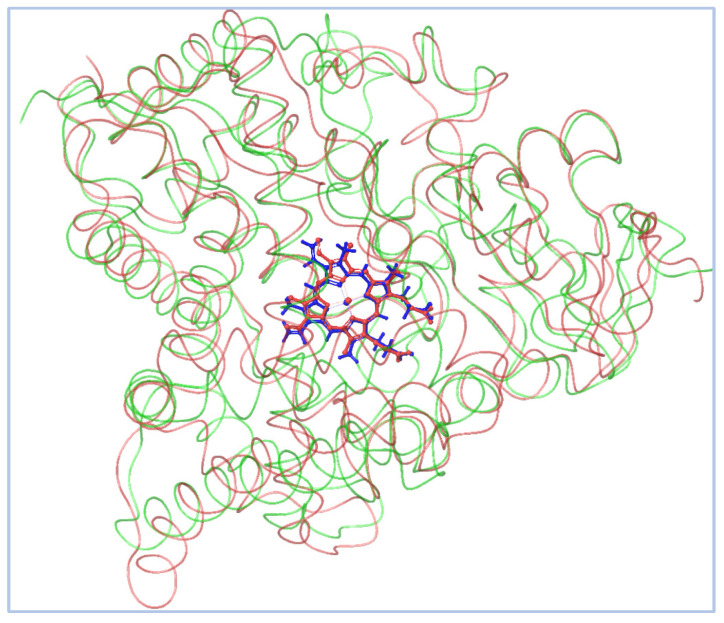
The homology-modeled *Madurella mycetomatis* CYP51 is shown in green, while the human CYP51 (PDB ID: 6UEZ) is depicted in faint red. The heme groups are highlighted in blue and red for the fungal and human enzymes, respectively, emphasizing the conserved positioning of the catalytic core despite structural divergence in peripheral regions.

To ensure inhibiter’s selectivity, the sequence alignment between the modeled CYP51 from *Madurella mycetomatis* and human CYP51 (PDB ID: 6UEZ) [63] (Figure 4) revealed a moderate degree of divergence. The overall sequence identity was 39%, with 56% similarity and 55% conserved residues. Previous studies have shown that the sequence identity of the CYP51 gene among various fungal species, when compared to the human ortholog, ranges from 36.5% to 93.9% [27]. In the present study, the CYP51 sequence from *Madurella mycetomatis* exhibited 39% identity to the human CYP51. These findings suggest that, while both enzymes share conserved structural motifs essential for function, significant differences exist at the amino acid level, particularly in regions potentially involved in ligand binding. This level of divergence may be exploited to design selective antifungal inhibitors that target the fungal enzyme without affecting the human counterpart. The N-terminal region (residues 1–50) showed limited identity, indicating variability in membrane anchoring or orientation. The central core (residues ~100–300), which typically houses the heme-binding and substrate recognition domains, exhibited higher conservation. Critical residues involved in the heme-thiolate coordination motif (FxxGxxxCxG), which contains a heme axial Cys ligand near the C-terminal end, were well-preserved, suggesting maintained catalytic function. However, significant divergence was observed in regions spanning residues 180–230 and 390–431. The latter segment (391–431) is notably absent in the human CYP51 crystal structure but present in the *Madurella mycetomatis* model, possibly forming a unique loop or extension. This insertion could represent a fungus-specific surface or pocket-lining structure. Structural alignment revealed notable variability near the substrate access channels and ligand-interacting loops, particularly in the F–G loop (residues 210–235), B′ region (120–140), and the C-terminal segment (390–431). These differences suggest an altered binding cavity topology, which may be exploited for selective targeting of the fungal enzyme while minimizing human off-target effects. The root-mean-square deviation (RMSD) between the aligned structures (Figure 5) of human CYP51 (PDB ID: 6UEZ) and the modeled *Madurella mycetomatis* CYP51 was 3.24 Å, indicating a moderate level of structural divergence. While the overall protein folds are similar, this value suggests noticeable conformational differences, particularly in loop regions or surface-exposed domains, which may contribute to differences in substrate access or ligand binding specificity.

### 2.2. Virtual Screening, Molecular Docking, and Binding Free Energy Calculations

**Figure 6:**
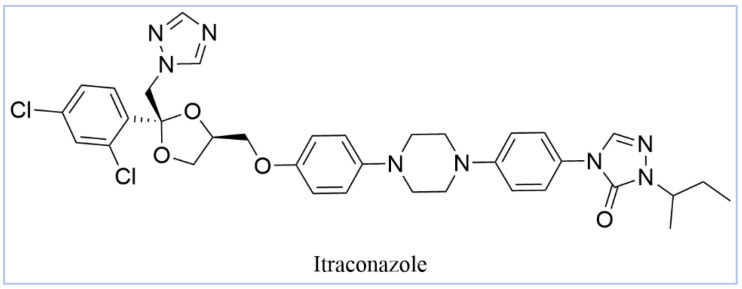
Chemical structure of itraconazole, the current treatment of eumycetoma.

Itraconazole (Figure 6), a triazole antifungal that inhibits CYP51, is the first-line treatment for eumycetoma. It is used alone or in combination with surgery, particularly in advanced cases [3,5,52]. While itraconazole targets the ergosterol biosynthesis pathway in *Madurella mycetomatis*, treatment outcomes can be variable, often requiring prolonged therapy [64]. Resistance and limited efficacy in some cases highlight the need for alternative therapeutic options [65,66].

In our study, we conducted a computational investigation of microbial-based natural compounds against our homology model of *Madurella mycetomatis* CYP51, using itraconazole as the reference drug. LigPrep (Schrödinger Release 2023-1: LigPrep, Schrödinger, LLC, New York, NY, USA, 2023) generated two ionization states of itraconazole (ionized and unionized), which were subjected to Extra Precision (XP) molecular docking against the homology model of *Madurella mycetomatis* CYP51, yielding docking scores of −5.952 and −6.565 kcal/mol, respectively (Table 1). 

**Figure 7:**
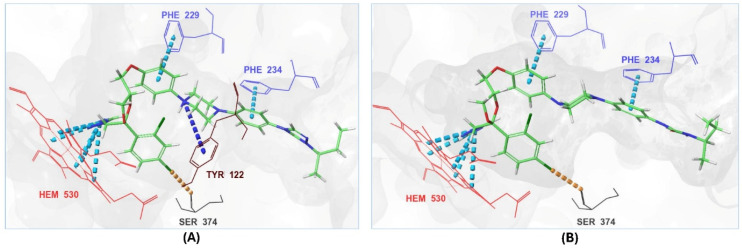
The 3D interactions of itraconazole’s ionized form (**A**) and unionized form (**B**) with the in-house homology model of the *Madurella mycetomatis* CYP51 binding site.

The ionized form interacted with CYP51 through a halogen bond with Ser374 (3.19 Å), a pi-cation interaction with Tyr122 (5.87 Å), and pi-pi stacking with Phe229 (4.74 Å), Phe234 (4.23 Å), and HEM530 (4.27 Å and 4.76 Å). Additionally, extensive hydrophobic interactions were observed with Leu91, Leu92, Ile121, Tyr122, Val124, Leu125, Val129, Phe130, Val135, Tyr136, Phe229, Phe234, Ala302, Met305, Ala306, Ile372, Ile375, Met376, Val397, Leu511, and Phe512. The unionized form exhibited a similar interaction profile, forming a halogen bond with Ser374 (3.21 Å) and pi-pi stacking with Phe229 (4.87 Å), Phe234 (3.88 Å), and HEM530 (4.28 Å and 4.77 Å). Hydrophobic interactions involved Tyr68, Leu91, Leu92, Ile121, Tyr122, Leu125, Phe130, Val135, Tyr136, Phe229, Phe234, Ala302, Met305, Ala306, Ile372, Ile375, Met376, Val397, Leu511, and Phe512 (Figure 7 and Supplementary Table S1).

**Figure 8:**
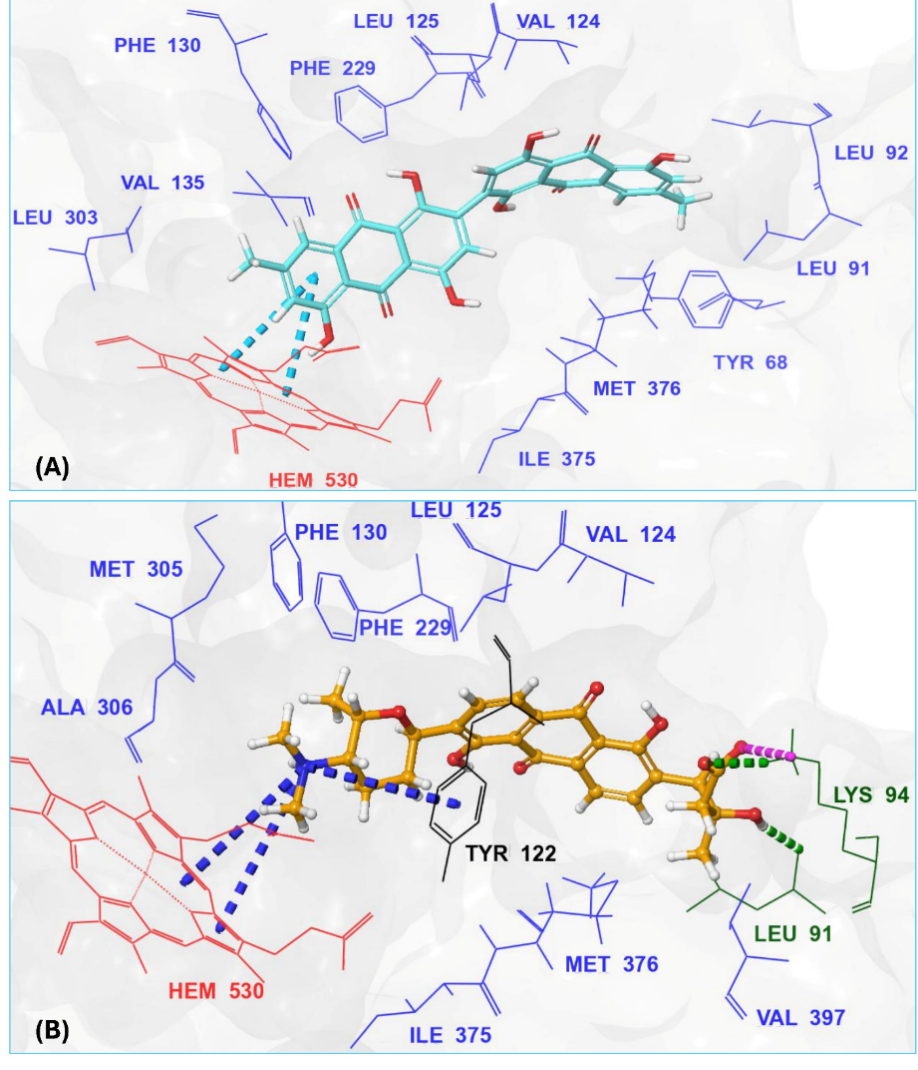
The 3D interaction diagrams of the two top-ranked, based on XP docking scores, microbial-based natural products, (**A**) NPA023185, and (**B**) NPA017021 showing their interactions with active site residues of the in-house homology model of *Madurella mycetomatis* CYP51.

With itraconazole’s docking results established, the next step involved subjecting all-natural compounds to high-throughput virtual (HTV) screening, followed by Standard Precision (SP) and XP docking against the *Madurella mycetomatis* CYP51 homology model. This revealed 234 compounds with superior docking scores to itraconazole, exhibiting diverse docking scores and interaction profiles at the enzyme active site. MM-GBSA calculations identified 34 compounds that outperformed itraconazole. However, a deeper analysis of binding interactions revealed that only 9 compounds interacted with the heme group, along with one or more key residues that play a role in positioning the heme within the *Madurella mycetomatis* CYP51 active site (Supplementary Table S1). These 9 compounds, therefore, merit further investigation, as they not only displayed better binding affinity than itraconazole but also interacted with both the heme and crucial residues involved in its positioning [27]. Among these compounds, NPA023185 exhibited the best docking score (−14.449 kcal/mol), followed by NPA017021 (−13.669 kcal/mol), indicating their strong potential as inhibitors (Table 1). Both compounds were appropriately positioned within the active site, binding to active site residues via different interactions, further supporting their inhibitory potential (Figure 8).

Other notable ligands, including NPA029353 (−11.649 kcal/mol), NPA029354 (−11.460 kcal/mol), NPA029352 (−11.716 kcal/mol), NPA020764 (−11.385 kcal/mol), and NPA020514 (−10.877 kcal/mol), demonstrated favorable docking scores, suggesting significant interactions with the active site. In contrast, NPA026108 (−10.299 kcal/mol) and NPA018887 (−9.626 kcal/mol) exhibited relatively weak binding strengths but were still superior to itraconazole, the reference standard, which displayed docking scores of −5.952 kcal/mol (ionized) and −6.565 kcal/mol (unionized) (Table 1). These findings highlight the enhanced binding of several natural products in comparison to this standard antifungal drug.

The interaction analysis further clarifies these docking results by revealing the molecular contacts between each ligand and the active site of the *Madurella mycetomatis* CYP51 homology model (Supplementary Table S1). NPA020764, which demonstrated strong binding (−11.385 kcal/mol), formed multiple hydrogen bonds with Gly69 (2.16 Å), Tyr136 (1.88 Å), Asp507 (2.47 Å), and HEM530 (2.43 Å), enhancing its interaction with key active site residues. Additionally, its salt bridge with Lys235 (3.31 Å) and HEM530 (4.8 Å) contributed to a low docking score, while pi-cation interaction with HEM530 (4.42 Å) facilitated coordination with the heme iron. Extensive hydrophobic interactions with Ile66, Tyr68, Met70, Leu91, Leu92, Ile121, Tyr122, Leu125, Tyr136, Phe229, Ile232, Pro231, Phe234, Ile372, Met376, Tyr508, Ala509, Leu511 and Phe512 further reinforced its binding, suggesting a strong inhibitory potential. Similarly, NPA017021, which exhibited a lower docking score (−13.669 kcal/mol), bound the active site through hydrogen bonding with Leu91 (2.11 Å) and Lys94 (2.24 Å), as well as a salt bridge with Lys94 (3.42 Å). It also formed pi-cation interactions with Tyr122 (5.74 Å) and HEM530 (6.0 Å and 6.47 Å), along with hydrophobic interactions with Leu91, Leu92, Ile121, Tyr122, Va124, Leu125, Phe130, Val135, Tyr136, Phe229, Met305, Ala306, Ile372, Met376, Val397, Phe234, Leu511 and Phe512. NPA023185, despite its more negative docking score (−14.449 kcal/mol), primarily relied on hydrophobic interactions with Tyr68, Leu91, Leu92, Ile121, Tyr122, Val124, Leu125, Phe130, Val135, Tyr136, Phe229, Phe234, Ala302, Leu303, Ala306, Ile372, Ile375, Met376, Leu511 and Phe512, with no hydrogen bonding or salt bridge formation. However, it engaged in pi-pi stacking with HEM530 (5.16 Å), which could influence the electronic environment of the heme group (Supplementary Table S1).

Other promising candidates, including NPA029352 (−11.716 kcal/mol), NPA029353 (−11.649 kcal/mol), and NPA029354 (−11.460 kcal/mol), exhibited various binding interactions. Specifically, NPA029352 formed a hydrogen bond with Ser510 (2.17 Å) and pi-cation interactions with HEM530 (4.56 Å and 5.27 Å), along with hydrophobic interactions with Tyr68, Leu91, Leu92, Ile121, Tyr122, Va124, Leu125, Phe130, Tyr136, Phe229, Phe234, Met305, Ala306, Ile372, Met376, Val397, Leu511 and Phe512. In comparison, NPA029353 formed pi-cation interactions with Tyr122 (5.89 Å) and HEM530 (5.86 Å and 6.43 Å), as well as pi-pi stacking with Phe234 (4.38 Å), but it lacked hydrogen bonds or salt bridges. It also formed hydrophobic interactions with a similar set of residues, including Tyr68, Leu91, Leu92, Ile121, Tyr122, Va124, Leu125, Phe130, Val135, Tyr136, Phe229, Phe234, Met305, Ala306, Ile372, Met376, Val397, Leu511 and Phe512. Lastly, NPA029354 engaged in pi-cation interactions with HEM530 (6.31 Å and 6.56 Å) but lacked the additional stabilizing forces seen in other ligands. It also exhibited hydrophobic interactions with residues common to other candidates, including Tyr68, Leu91, Leu92, Ile121, Tyr122, Va124, Leu125, Phe130, Tyr136, Phe229, Phe234, Met305, Ala306, Ile372, Met376, Val397, Leu511 and Phe512. NPA020514 (−10.877 kcal/mol) exhibited a hydrogen bond with Ile121 (1.76 Å) and pi-pi stacking interactions with HEM530 (4.69 Å and 5.36 Å). Furthermore, it formed hydrophobic interactions with Tyr68, Leu91, Leu92, Ile121, Tyr122, Val124, Leu125, Phe130, Val135, Tyr136, Phe229, Phe234, Ala306, Ile372, Met376, and Leu511, which collectively contributed to a moderate binding strength. In contrast, NPA026108 (−10.299 kcal/mol) formed a hydrogen bond with Lys94 (2.48 Å) and a pi-cation interaction with HEM530 (6.23 Å). However, its binding was primarily driven by hydrophobic interactions with Leu91, Leu92, Ile121, Tyr122, Val124, Leu125, Phe130, Tyr136, Phe229, Phe234, Met305, Ile372, Ile375, Met376, Val397, Leu511, and Phe512. Lastly, NPA018887 (−9.626 kcal/mol) formed a hydrogen bond with Leu91 (2.14 Å) and engaged in pi-pi stacking with HEM530 (5.43 Å). Additionally, it interacted hydrophobically with Tyr68, Leu91, Leu92, Ile121, Tyr122, Val124, Leu125, Phe130, Val135, Tyr136, Phe234, Ala302, Met305, Ala306, Ile372, Met376, Val397, Leu511, and Phe512 (Supplementary Table S1). Despite these interactions, its binding strength appeared weaker in comparison.

**Figure 9:**
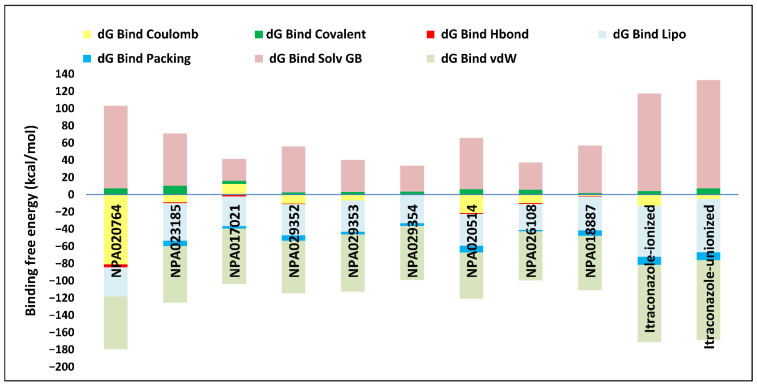
Detailed MM-GBSA binding free energy contributions for the complexes of the top-ranked microbial-based natural products and itraconazole (in both ionized and unionized forms) with the in-house homology model of *Madurella mycetomatis* CYP51.

Overall, these natural products exhibited varying but superior docking scores compared to itraconazole (Table 1), along with extensive binding interactions with active site residues, including some interactions with the heme group (Supplementary Table S1). These findings suggest they are promising *Madurella mycetomatis* CYP51 inhibitors. That said, docking scores provide an initial estimation of binding interactions; however, they often produce false positives due to their inability to account for dynamic receptor behavior, solvation effects, and entropy [67]. To address these limitations, further validation is performed using MM-GBSA binding energy calculations or MD simulations [55]. For instance, the docking results of the current study indicated that the unionized form of itraconazole had a slightly more favorable docking score (−6.565 kcal/mol) than the ionized form (−5.952 kcal/mol). Nonetheless, MM-GBSA calculations revealed that the ionized form had a significantly more favorable binding energy, with a value of −54.07 kcal/mol compared to −36.07 kcal/mol for the unionized form (Table 1). This difference can be explained by the fact that various energy components contribute to the total binding free energy in MM-GBSA calculations, reflecting different interaction forces between the ligand and the protein. Specifically, dG Bind Coulomb represents electrostatic interactions; thus, a more negative value indicates strong favorable attraction, whereas a positive value suggests repulsion. Similarly, dG Bind Covalent is relevant when a covalent bond forms between the ligand and the protein. In this case, a negative value signifies stable binding, whereas it is typically zero if no covalent interaction occurs. Moreover, dG Bind Hbond measures hydrogen bonding contributions. Here, more negative values reflect strong hydrogen bonds that enhance binding stability. Conversely, weak or nonexistent hydrogen bonds result in values closer to zero or even positive. Likewise, dG Bind Lipo quantifies hydrophobic interactions. In this context, negative values suggest strong binding within hydrophobic pockets, whereas positive values indicate poor fitting or exposure of hydrophobic regions to a polar environment. Furthermore, dG Bind Packing also referred to as the pi-pi packing correction, accounts for pi-pi stacking interactions between aromatic rings in the ligand and protein. A more negative value suggests strong pi-pi interactions; in contrast, a less negative or positive value indicates weak or unfavorable stacking due to suboptimal ring orientation or steric clashes. Additionally, dG Bind Solv GB accounts for solvation energy. In this regard, a positive value indicates an unfavorable desolvation penalty due to excessive loss of stabilizing solvent interactions, whereas a negative value suggests a favorable solvation contribution. Finally, dG Bind vdW represents van der Waals interactions. Here, highly negative values indicate strong non-bonded dispersion forces and excellent shape complementarity, while less negative or positive values suggest weaker steric interactions and poor ligand fitting within the binding pocket [68]. In our study, the MM-GBSA calculations revealed that the binding energy components for ionized and unionized itraconazole differed in several key aspects. For the ionized form of itraconazole, the Coulombic interactions (dG Bind Coulomb) contributed significantly to the binding, with a value of −13 kcal/mol, indicating favorable electrostatic interactions with the protein. In contrast, the unionized form exhibited a less negative value of −5 kcal/mol, suggesting weaker electrostatic interactions (Figure 9).

Both ionized and unionized itraconazole showed no significant contribution from hydrogen bonding (dG Bind Hbond), with a value of 0 kcal/mol for both forms, indicating that hydrogen bonding did not play a major role in their binding affinity. The van der Waals (dG Bind vdW) interactions were favorable for both forms, with values of −90 kcal/mol for the ionized form and −93 kcal/mol for the unionized form, indicating strong non-covalent interactions between the ligand and the protein. Notably, the unionized form showed slightly better van der Waals interactions (more negative value), but this was not enough to outweigh other factors. Regarding lipophilic interactions (dG Bind Lipo), the ionized form had a favorable value of −59 kcal/mol, while the unionized form showed an even more favorable value of −62 kcal/mol, suggesting that the unionized form had slightly stronger hydrophobic interactions. Solvation effects, however, showed a different trend: the ionized form had a solvation energy (dG Bind Solv GB) of 113 kcal/mol, while the unionized form had a slightly higher value of 126 kcal/mol. Positive solvation energies indicated a penalty, meaning that solvation had a less favorable effect on the binding of the unionized form compared to the ionized form. Finally, the packing energy (dG Bind Packing) was similar for both forms, with a value of −9 kcal/mol, suggesting that the structural arrangement within the binding site was comparable for both ionized and unionized forms. In summary, the ionized itraconazole demonstrated more favorable overall binding energy, primarily due to stronger Coulombic and lipophilic interactions, as well as a less penalizing solvation energy compared to the unionized form. This highlights that while the docking score serves as a useful initial screening tool, binding energy, as demonstrated in the case of ionized itraconazole, is typically regarded as a more accurate measure for assessing binding affinity [69].

Thus, refining docking results with post-docking analyses, such as MM-GBSA, is essential for a more comprehensive evaluation, as it accounts for solvation effects and entropic contributions. Accordingly, incorporating MM-GBSA calculations reduces the risk of over-reliance on docking scores, ensuring a more accurate representation of the true binding potential of drug candidates [70]. In this context, the protein-ligand complexes of the investigated natural compounds showed varying binding energies. Of these, 34 compounds exhibited stronger affinities for the *Madurella mycetomatis* CYP51 homology model than itraconazole, as determined by MM-GBSA calculations. However, as mentioned earlier, only 9 compounds showed promising binding interactions at the CYP51 active site (Supplementary Table S1), with NPA020764 exhibiting the strongest binding (−76.66 kcal/mol), followed by NPA029353 (−72.39 kcal/mol) and NPA029354 (−65.62 kcal/mol), suggesting these ligands may have high affinity for *Madurella mycetomatis* CYP51 and are promising candidates for further investigation as potential inhibitors (Table 1 and Figure 9). Moderate binding affinities were seen with NPA017021 (−62.35 kcal/mol), NPA026108 (−62.10 kcal/mol), and NPA029352 (−58.89 kcal/mol), which may warrant further optimization or experimental validation. Weaker interactions were observed with NPA020514 (−55.05 kcal/mol), NPA023185 (−54.51 kcal/mol), and NPA018887 (−54.34 kcal/mol), indicating these ligands may have lower potential as inhibitors but could still be useful for structural modifications or screening [71]. As for itraconazole, the ionized form (−54.07 kcal/mol) demonstrated moderate binding, while the unionized form (−36.07 kcal/mol) exhibited the weakest binding, suggesting that ionization plays a crucial role in its affinity for the target (Table 1). While NPA020764 and NPA029353 stand out as the most promising natural ligands for *Madurella mycetomatis* CYP51 inhibition, exhibiting stronger binding energies than other natural compounds and itraconazole, a more detailed breakdown and analysis of their binding profiles are essential to gain a comprehensive understanding of their interactions with key active site residues. Such analysis will help identify specific binding features and interaction patterns, which are crucial for optimizing their potency and selectivity. This deeper understanding will provide valuable insights that can guide future structural optimizations, ultimately enhancing the efficacy of these natural ligands as potential therapeutic agents for eumycetoma treatment [72].

As previously discussed, MM-GBSA calculations provide valuable insights into drug-target interactions. In this study, we further analyze the binding energy breakdown of the top-ranked natural product complexes with CYP51, focusing on key energetic contributions, including Coulombic (electrostatic) interactions, covalent interactions, hydrogen bonding, lipophilic interactions, packing effects, solvation energy, and van der Waals forces. Coulombic interactions showed significant variability across all compounds. NPA020764 exhibited the strongest stabilizing Coulombic interaction (−81 kcal/mol), while most other compounds, including itraconazole (−13 kcal/mol), displayed weaker electrostatic contributions. This suggests that electrostatic interactions were critical in stabilizing certain natural products within the active site. Covalent interactions, however, had minimal contributions across all compounds, with values ranging from 2 to 10 kcal/mol. This indicates that covalent bonding did not significantly affect the overall binding affinity in this set of molecules. Hydrogen bonding contributions were also relatively low, with most compounds showing values between −1 and −4 kcal/mol. Only NPA020764 exhibited a slightly stronger hydrogen bond contribution (−4 kcal/mol). These findings suggest that hydrogen bonding did not play a dominant role in ligand binding. In contrast, lipophilic interactions (dG Bind Lipo) were substantial across all compounds, with values ranging from −30 to −59 kcal/mol. Itraconazole displayed the highest lipophilic interaction (−59 kcal/mol), which reinforces its strong affinity for the hydrophobic regions of CYP51. Among the natural products, NPA023185 (−44 kcal/mol) showed the highest lipophilic contribution, while NPA0260108 (−30 kcal/mol) exhibited the lowest. Packing contributions, which reflect steric interactions, were relatively minor, with most compounds displaying values between −1 and −9 kcal/mol. This suggests that packing effects did not significantly drive binding. On the other hand, solvation energy (dG Bind Solv GB) exhibited the most pronounced variations. Itraconazole had the highest solvation penalty (113 kcal/mol), reflecting the high cost of desolvation upon binding. Comparatively, NPA020764 (96 kcal/mol) also experienced a substantial desolvation penalty, indicating that it required considerable structural adaptation to fit into the binding pocket. The lowest desolvation penalty was observed with NPA017021 (26 kcal/mol). Finally, van der Waals (vdW) interactions played a crucial role in stabilizing the complexes. Itraconazole exhibited the strongest vdW contribution (−90 kcal/mol), emphasizing its high affinity for CYP51. Among the natural products, NPA029353 (−67 kcal/mol) and NPA023185 (−66 kcal/mol) also displayed quite strong vdW interactions, suggesting that these compounds engaged in significant non-covalent interactions with the enzyme.

This breakdown highlights the key forces governing ligand binding to *Madurella mycetomatis* CYP51. While Coulombic interactions and van der Waals forces were crucial for stabilizing some of the natural products, lipophilic interactions emerged as a dominant factor in ligand binding. The high solvation penalties for certain compounds indicate that structural modifications could enhance their drug-like properties. Overall, these microbial-derived natural products exhibited promising characteristics as potential CYP51 inhibitors, with select candidates displaying comparable or superior interactions to itraconazole in specific energy components.

### 2.3. In Silico ADMET Profiling

ADMET plays a critical role in drug discovery by assessing how a drug behaves in the body and its potential for adverse effects. It helps identify compounds with favorable pharmacokinetic profiles and safety characteristics early in development, reducing the risk of failure in later stages. Methods to evaluate ADMET include in silico predictions using computational tools [56,73], as well as experimental approaches such as in vitro testing and animal studies [74]. Combining these methods optimizes drug candidates and enhances the chances of successful development [75].

In QikProp [76], a tool for assessing the ADMET properties of small molecules, the term “stars” measures how well a molecule’s properties align with those of known drugs. This metric indicates the number of molecular properties that fall outside the recommended range for 95% of drugs in QikProp’s database [77]. A molecule with 0 stars is considered ideal, meaning all its calculated properties fall within the typical drug-like range. Conversely, a higher star count (>5) suggests that more properties deviate from this range, potentially reducing the molecule’s likelihood of success as a drug candidate. The star rating system is valuable for quickly identifying molecules with suboptimal physicochemical properties that may pose challenges in drug development [78]. To accelerate drug discovery, candidates with fewer stars are prioritized in virtual screening as they require minimal refinement, whereas those with higher star counts may undergo optimization to enhance their properties or be excluded to streamline the selection of promising drug candidates [79].

The QikProp stars analysis of the tested compounds revealed varying degrees of drug-likeness (Table 2). Compounds NPA017021, NPA020514, NPA029352, NPA029353, and NPA029354 exhibited 0 stars, indicating optimal drug-like properties, as all calculated parameters fall within the typical range of known drugs. NPA018887, with 1 star, has only one property outside the ideal range, requiring minimal optimization. Similarly, NPA026108, with 2 stars, has two flagged properties, suggesting a slightly greater need for refinement. In contrast, NPA023185, with 4 stars, showed deviations in four properties, indicating potential challenges in drug development. Notably, NPA020764, with 12 stars, had a significant number of properties outside the acceptable range (0–5), making it a poor candidate for drug-likeness without major structural modifications [71].

The physicochemical properties of a drug, such as its size, solubility, and lipophilicity, play a vital role in determining its therapeutic efficacy [80]. The QikProp analysis showed that the molecular weights of the compounds ranged from 433.5 (NPA029352) to 624.8 (NPA020764), all falling within the acceptable range of 130 to 725, indicating an appropriate size for drug-like molecules. The solvent-accessible surface area (SASA) values were within the permissible range of 300 to 1000, except for compound NPA020764, which exceeded the limit with a value of 1218.3. This suggests inadequate interaction of NPA020764 with the aqueous environment, contrary to the other compounds. A drug’s ability to permeate cell membranes is a key determinant of its absorption rate and extent in the human body, directly affecting its bioavailability [81]. The polar surface area (PSA) is widely used in drug discovery and development as it correlates with a drug’s ability to penetrate cell membranes [82]. Compounds NPA023185 and NPA020764 exhibited PSA values of 219.9 and 278.1, respectively, exceeding the accepted range of 7 to 200. In contrast, the PSA values of other compounds ranged from 83.4 (NPA029352) to 172.6 (NPA020514), all falling within the acceptable range (Table 2).

The hydrogen bond donor (donorHB) values were 0 for NPA023185, 1 for NPA017021, NPA018887, NPA020514, and NPA029353, and 2 for NPA026108, NPA029352, and NPA029354, all within the permissible range of 0–6. However, NPA020764 exhibited a donorHB value of 8.25, exceeding this range, which may pose challenges for membrane permeability and bioavailability. In contrast, the hydrogen bond acceptor (accptHB) values ranged from 6.5 for NPA023185 to 15.75 for NPA020764, all within the acceptable range of 2–20 (Table 2).

The percent human oral absorption values categorize the compounds into high (>80%), moderate (25–80%), and poor (<25%) absorption groups, offering insights into their bioavailability [83]. Compounds NPA018887 (92.3%), NPA029352 (87.9%), NPA029353 (85.4%), and NPA029354 (84.5%) exhibited high absorption, indicating favorable oral bioavailability and suitability for further development. Moderate absorption was observed in NPA017021 (40.1%), NPA020514 (59.0%), and NPA026108 (49.5%), suggesting that these compounds may benefit from optimization to enhance their bioavailability. Conversely, NPA020764 (0.0%) exhibited no measurable absorption, while NPA023185 (26.8%) demonstrated nearly poor absorption, making these drugs less viable drug candidates without substantial modifications [71]. Prioritizing compounds with high absorption could streamline the drug discovery process by focusing on those requiring minimal refinement (Table 2) [50].

QPPCaco (a model for gut-blood barrier) is a predicted measure of a compound’s permeability through Caco-2 cells, expressed in nanometers per second (nm/s). It estimates how well a drug can pass through the intestinal wall, which is crucial for its oral absorption and bioavailability. This computational metric plays an important role in drug discovery by evaluating the potential of a compound to permeate the intestinal lining. In our study, the QPPCaco values for the tested compounds revealed distinct intestinal permeability characteristics, influencing their bioavailability potential [84]. NPA018887 exhibited high permeability with a QPPCaco value of 594.5, exceeding the threshold of 500, which indicates significant absorption potential. Compounds NPA029352, NPA029353, and NPA029354 fell within the accepted range for moderate permeability (25 to 500), with values of 242.7, 128.8, and 114.7, respectively. In contrast, the remaining compounds demonstrated low permeability, with QPPCaco values below 25, ranging from 0 for NPA020764 to 21.2 for NPA026108. These results underscore varying degrees of intestinal permeability, which directly correlate with the bioavailability potential of the compounds, suggesting that only a subset of these may have favorable absorption profiles (Table 2) [85].

Plasma protein binding is a key factor influencing both the pharmacokinetics and pharmacodynamics of drugs. Although high plasma protein binding can prolong a drug’s duration of action, it may also decrease the concentration of free (active) drug in circulation, potentially reducing its therapeutic effectiveness [86]. The QPlogKhsa (a Schrödinger’s QSAR-based descriptor for human serum albumin binding) analysis, which has an acceptable range of −1.5 to 1.5 [87], demonstrated that all compounds fell within the optimal range, with values spanning from −1.14 for NPA020764 to 0.86 for NPA018887. This balance between free (active) and bound forms helps maintain sustained therapeutic levels in circulation, potentially enhancing efficacy, reducing dosing frequency, and improving their suitability as drug candidates (Table 2) [50].

In drug development, the inhibition of the human ether-a-go-go-related gene (hERG) channel by small molecules is a major concern in the pharmaceutical industry [88]. The QPlogHERG values for the compounds NPA017021, NPA018887, NPA020514, NPA020764, NPA023185, NPA026108, NPA029352, NPA029353, and NPA029354 were as follows: −4.20, −5.43, −6.41, −4.10, −6.30, −5.66, −6.18, −6.15, and −6.55, respectively. Compounds NPA017021 and NPA020764, with values above −5.0, suggest a lower risk for hERG inhibition and are considered safer in terms of cardiac toxicity. In contrast, compounds NPA018887, NPA026108, and NPA023185, with values near or slightly below −5.0, indicate moderate concern for hERG inhibition, warranting further investigation. The remaining compounds, NPA020514, NPA029352, NPA029353, and NPA029354, all showed QPlogHERG values well below −5.0, suggesting a high likelihood of hERG channel inhibition and a significant risk for cardiac toxicity, thus requiring more rigorous testing to confirm their safety profile (Table 2).

Metabolic stability is a crucial parameter in drug development, as it directly impacts the pharmacokinetic characteristics of a compound, including its bioavailability, half-life, and potential for adverse effects [89]. The accepted range for the number of metabolites a compound can generate is typically 1 to 8, as exceeding this range can indicate excessive biotransformation, which may alter the compound’s efficacy or lead to the formation of undesirable metabolites. In this analysis, NPA018887 has been identified as the most metabolically stable compound, with only 1 metabolite, which suggests minimal biotransformation and, therefore, a higher likelihood of maintaining its intended pharmacological activity. On the other hand, NPA029352, NPA029353, and NPA029354 generated 6 metabolites each. While this falls within the acceptable range, it suggests a moderate degree of metabolic turnover, which could lead to reduced drug efficacy over time or the potential formation of unwanted metabolites. NPA020514 produced 5 metabolites, while NPA017021 and NPA023185 each generated 8 metabolites. While NPA020514, NPA029352, NPA029353, and NPA029354 are still within the acceptable range, NPA017021, and NPA023185, with eight metabolites, are approaching the upper limit, which might necessitate further examination of their metabolic profile to ensure it remains effective and safe. NPA020764 and NPA026108, however, both exceeded the upper limit with 10 and 9 metabolites, respectively, indicating significant metabolic instability (Table 2). Such high metabolic turnover could lead to rapid elimination, reduced therapeutic efficacy, or the generation of metabolites that might be toxic or interfere with the desired therapeutic outcomes [90]. These compounds would likely need to undergo structural modifications or optimization to improve their metabolic stability and maintain a favorable pharmacokinetic profile. In conclusion, the compounds within the accepted range of 1 to 8 metabolites are generally preferred, as they are more likely to exhibit desirable pharmacokinetic properties, whereas those exceeding this range require additional modifications and testing [71].

Lipinski’s Rule of 5 is a set of guidelines used to predict the oral bioavailability of potential drug compounds. The rule suggests that for a compound to be a good oral drug candidate, it should have a molecular weight of less than 500 daltons, a LogP (partition coefficient) of less than 5, no more than 5 hydrogen bond donors (such as NH or OH groups), and no more than 10 hydrogen bond acceptors (such as oxygen or nitrogen atoms). These parameters are believed to correlate with a compound’s ability to be absorbed into the bloodstream when taken orally [91]. While these rules are generally useful, they are not absolute, and some compounds that do not strictly follow them can still be effective drugs [92]. In our study, all compounds adhered to Lipinski’s Rule of Five, which permits up to four violations, with most compounds showing no violations, indicating full compliance. Notably, NPA018887, NPA023185, and NPA020764 each presented 1, 2, and 3 violations, respectively, all of which remain within the allowed limit. Specifically, NPA018887 exceeded the molecular weight limit (MW = 522.589), NPA023185 exceeded the molecular weight limit (MW = 538.466) and lacked hydrogen bond donor groups, and NPA020764 exceeded the molecular weight limit (MW = 624.773), the number of hydrogen bond donor groups (donorHB = 8.25), and the number of hydrogen bond acceptor groups (accptHB = 15.75) (Table 2). Despite these violations, each compound remains within the permissible limits of Lipinski’s Rule and is considered drug-like and suitable for further development.

Jorgensen’s Rule of Three in QikProp focuses on predicting the oral bioavailability of drug candidates by evaluating three key criteria. These are: (1) a predicted solubility (QPlogS) greater than −5.7, indicating that the compound is sufficiently soluble for absorption; (2) a predicted permeability (QPPCaco) greater than 22 nm/s, which suggests the compound can effectively pass through the intestinal membrane; and (3) fewer than 7 primary metabolites, indicating a simpler metabolic profile that reduces the risk of complex metabolism and potential toxicity [93]. Compounds that violate fewer of these rules are more likely to be orally bioavailable, meaning they have a higher chance of being absorbed and utilized effectively when taken by mouth. Violations of these criteria may indicate challenges in solubility, permeability, or metabolism, which can hinder the drug’s potential for oral administration. All compounds in our study satisfied Jorgensen’s Rule of Three, which allows up to three violations, suggesting a higher likelihood of oral availability. NPA029352 and NPA029353 showed no violations, while NPA020514, NPA029354, and NPA018887 each had 1 violation. NPA017021, NPA026108, and NPA020764 each had 2 violations, and NPA023185 had 3 violations. Specifically, NPA020514 violated QPPCaco (17.05), NPA029354 violated QPlogS (−5.724), and NPA018887 violated QPlogS (−6.038). NPA017021, NPA026108, and NPA020764 violated the number of primary metabolites (8, 9, and 10, respectively). Additionally, these compounds also violated QPPCaco (2.354, 21.165, and 0.035, respectively) (Table 2). Despite these violations, all compounds remain within the acceptable limit, supporting their potential for oral bioavailability [93].

The predicted brain/blood partition coefficient (QPlogBB) [94], with an accepted range of −3.0 to 1.2, was evaluated for all compounds. Most of the compounds fell within the acceptable range, with values ranging from −2.96 for NPA020514 to −0.26 for NPA029352, suggesting moderate to favorable brain penetration. However, two compounds, NPA020764 and NPA023185, showed significantly lower values of −8.83 and −4.08, respectively, which are well outside the acceptable range. These results indicate that NPA020764 and NPA023185 have poor blood-brain barrier penetration and limited potential for central nervous system activity (Table 2).

Based on the predicted ADMET properties, NPA020764 exhibited the poorest ADMET properties, violating 12 key drug-like parameters and exceeding the most commonly accepted thresholds. Consequently, it was excluded from further analysis. Likewise, NPA017021 showed 2 violations of drug-like properties and reached the upper limit for predicted metabolites. With an oral bioavailability of only 40%, it was removed from further evaluation. Similarly, NPA023185 was excluded due to 7 drug-like property violations, including 4 stars nearing the maximum threshold and poor predicted oral bioavailability. Additionally, it failed three parameters of Jorgensen’s Rule of Three—the maximum allowable limit—as well as two criteria of Lipinski’s Rule of Five, where the threshold is 4 violations. Along the same lines, NPA020514 exhibited 3 violations of drug-like properties and only moderate oral bioavailability, warranting its exclusion from further screening. In a comparable manner, NPA026108 failed 4 drug-like properties while displaying moderate oral bioavailability, leading to its removal from subsequent evaluations. Although NPA018887 violated only 2 drug-like properties and demonstrated good oral bioavailability, it exhibited the lowest binding affinity among the investigated natural compounds. As a result, it was not advanced to the next assessment phase. By contrast, NPA029352, NPA029353, and NPA029354 exhibited the most favorable ADMET profiles, each violating only a single drug-like property while maintaining good oral bioavailability (Table 2). Notably, these compounds demonstrated superior binding affinities compared to itraconazole (Table 1) and were thus selected for MD simulations.

Human CYP51, also known as lanosterol 14α-demethylase, is a critical enzyme in the cholesterol biosynthesis pathway. However, inhibiting this enzyme can be toxic, making selective targeting essential in drug development [95]. Azole antifungals can inadvertently inhibit human CYP51, leading to disruptions in cholesterol and steroid hormone synthesis, and resulting in adverse side effects [96]. To assess potential off-target effects, the most promising microbial natural products identified in this study were also evaluated against human CYP51 (PDB ID: 6UEZ) [63]. Interestingly, docking scores and binding energy calculations indicate that the natural products NPA029352, NPA029353, and NPA029354 are less likely to inhibit human CYP51 compared to their binding to *Madurella mycetomatis* CYP51. Specifically, the docking scores for human CYP51 were −8.719, −6.156, and −8.776 kcal/mol for NPA029352, NPA029353, and NPA029354, respectively, while the corresponding scores for *Madurella mycetomatis* CYP51 were significantly lower: −11.716, −11.649, and −11.460 kcal/mol. Similarly, binding energies for the human enzyme were −41.12, 4.86, and −19.05 kcal/mol, in contrast to −58.89, −72.39, and −65.62 kcal/mol for the fungal counterpart. Taken together, these findings suggest that the identified natural products are promising candidates, as they demonstrated higher binding affinity toward fungal CYP51 compared to the human homolog. This selectivity is crucial, as the ideal antifungal agent should be a potent inhibitor of fungal CYP51 while leaving the human CYP51 unaffected [95].

### 2.4. Molecular Dynamics (MD) Simulations

The MD simulations of the *Madurella mycetomatis* CYP51 homology model offer valuable insights into the protein’s structural stability and flexibility in both its apo form and when bound to itraconazole and three other ligands (NPA029352, NPA029353, and NPA029354). Stability, assessed through protein-ligand root mean square deviation (PL-RMSD) metrics, reveals the behavior of these complexes over time, while flexibility, evaluated using protein-ligand root mean square fluctuation (PL-RMSF) metrics, captures the average fluctuations of CYP51 residues during the simulation [97].

**Figure 10:**
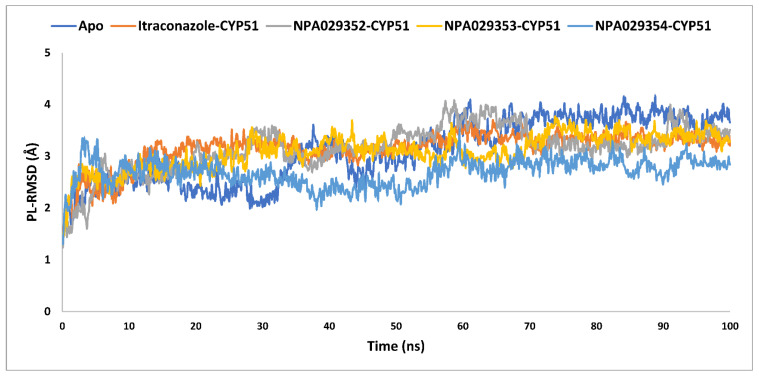
The PL-RMSD analysis of the apo-protein of the in-house homology model of *Madurella mycetomatis* CYP51 and its complexes with itraconazole, NPA029352, NPA029353, and NPA029354, as monitored throughout the MD simulation trajectories.

The average PL-RMSD values for the apo form, itraconazole, NPA029352, NPA029353, and NPA029354 were 3.1 Å, 3.2 Å, 3.1 Å, 3.1 Å, and 2.7 Å, respectively (Table 3 and Figure 10). These results indicate that, while the apo form and most ligand-bound complexes exhibited moderate fluctuations, NPA029354 stood out with the lowest average PL-RMSD, suggesting the strongest and most stable binding within the active site during the simulation. The maximum PL-RMSD values further corroborate this trend, with the apo form showing the highest deviation at 4.2 Å, while itraconazole, NPA029352, NPA029353 and NPA029354 exhibited maximum values of 3.7 Å, 4.1 Å, 3.8 Å and 3.5 Å, respectively. These data highlight that NPA029354 had the smallest maximum deviation, indicating it maintained the most consistent and stable binding throughout the simulation. In contrast, NPA029352, with a maximum PL-RMSD closer to the apo form, displayed slightly less stability compared to NPA029354. The minimum PL-RMSD values further reflect a tight interaction between the ligands and the binding site. While the apo form and itraconazole showed minimum values of 1.5 Å, NPA029354 demonstrated even tighter binding, with a value of 1.3 Å. These findings indicate that NPA029354 not only bound stably but also maintained a strong and consistent interaction with the binding pocket throughout the simulation. Similarly, the minimum values for NPA029352 and NPA029353 were 1.2 Å and 1.4 Å, respectively. In summary, NPA029354 emerges as the most promising ligand, exhibiting the lowest average and maximum deviations, coupled with tight minimum binding, suggesting superior stability and interaction within the CYP51 active site compared to the other tested ligands. This makes it a compelling candidate for further investigation as a potential inhibitor of *Madurella mycetomatis* CYP51.

**Figure 11:**
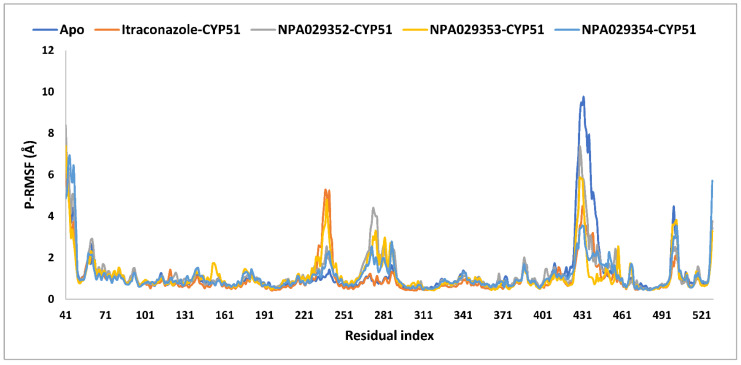
The P-RMSF analysis of the apo-protein of the in-house homology model of *Madurella mycetomatis* CYP51 and its complexes with itraconazole, NPA029352, NPA029353, and NPA029354, as monitored throughout the MD simulation trajectories.

The average protein root mean square fluctuations (P-RMSF) were comparable across all systems, with values ranging from 1.0 Å (itraconazole-bound) to 1.2 Å (apo, NPA029353-bound, and NPA029352-bound), indicating that itraconazole induced greater overall stabilization of the protein (Table 3 and Figure 11). Maximum P-RMSF values highlight significant differences, with the apo form showing the highest fluctuation (9.7 Å), itraconazole showing the lowest (6.3 Å), and the other ligands displaying intermediate values (8.4 Å for NPA029352, 7.4 Å for NPA029353, and 6.9 Å for NPA029354), suggesting varying degrees of structural stabilization. The minimum P-RMSF values (0.4–0.5 Å) were consistent across all systems, reflecting the stability of core regions unaffected by ligand binding. Overall, itraconazole demonstrated the strongest stabilizing effect, consistent with its established antifungal efficacy, while NPA029354 showed greater stabilization compared to NPA029352 and NPA029353, highlighting its potential as a promising drug candidate. These findings emphasize the differential effects of ligand binding on protein dynamics and underscore the importance of NPA029354 for further investigation.

**Figure 12:**
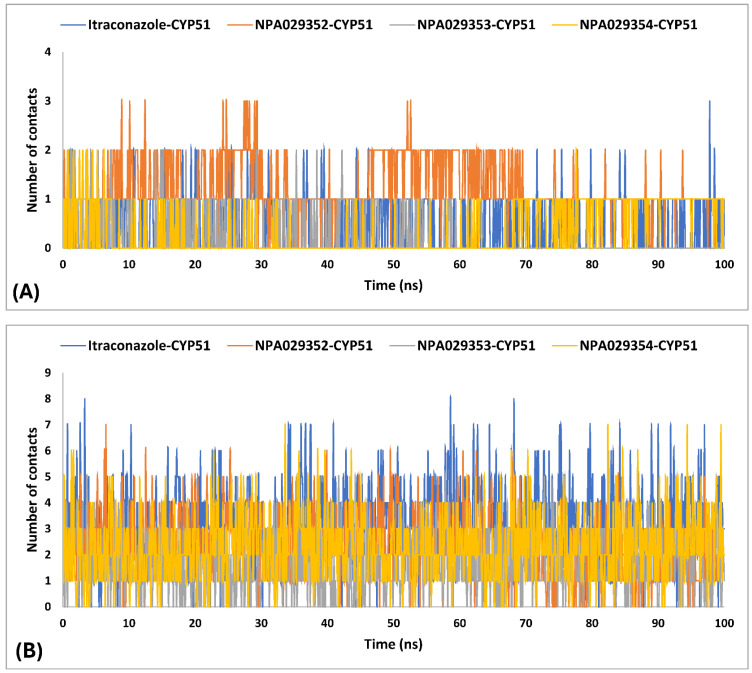
Number of H-bonds (**A**) and hydrophobic (**B**) contacts established during the entire MD simulations run for the complexes of the in-house homology model of *Madurella mycetomatis* CYP51 with itraconazole, NPA029352, NPA029353, and NPA029354, as monitored throughout the MD simulation trajectories.

In MD simulations of ligand-protein complexes, hydrogen bonds, and hydrophobic contacts play crucial roles in stability, binding affinity, and specificity. Hydrogen bonds enhance ligand binding through electrostatic interactions, contributing to stability and selectivity, while their persistence over time indicates ligand retention. Hydrophobic interactions, on the other hand, drive ligand association by minimizing water exposure, reinforcing structural integrity, and enhancing binding affinity through van der Waals forces. Analyzing these interactions in MD simulations helps assess ligand stability, solvent effects, and overall binding efficiency, providing insights into drug design and optimization [98,99]. The MD simulations findings for the interactions of itraconazole and three other ligands (NPA029352, NPA029353, and NPA029354) with the *Madurella mycetomatis* CYP51 homology model revealed differences in hydrogen bond (H-bond) contacts (Table 3 and Figure 12). On average, itraconazole formed 0.5 H-bond contacts, while NPA029352, NPA029353, and NPA029354 showed averages of 1.2, 0.2 and 0.4 contacts, respectively. The maximum H-bond contacts observed were 3.0 for itraconazole and NPA029352 and 2.0 for both NPA029353 and NPA029354. Despite these variations in average and maximum contacts, all four compounds demonstrated a minimum of 0 H-bond contacts, indicating that there were instances during the simulation where no hydrogen bonding occurred. These findings suggest that NPA029352 exhibited the most consistent potential for H-bond formation, as reflected in its higher average and maximum values, which may contribute to its stability and binding efficacy with the CYP51 homology model.

The MD simulations findings on the *Madurella mycetomatis* CYP51 homology model revealed notable differences in hydrophobic interactions among the studied ligands (Table 3 and Figure 12). Itraconazole exhibited the highest average hydrophobic contacts, consistent with the known binding mechanism of azole drugs [100], at 3.1, followed by NPA029352, NPA029354, and NPA029353 with averages of 2.4, 2.3, and 1.7 contacts, respectively. The maximum hydrophobic contacts were observed at 8.0 for itraconazole, while NPA029352, NPA029353, and NPA029354 reached the maximum of 7.0, 6.0, and 7.0 contacts, respectively. Notably, the minimum hydrophobic contacts for all compounds were consistently 0 throughout the simulations, indicating intermittent loss of hydrophobic interactions during the dynamics. These results suggest that itraconazole maintained stronger and more frequent hydrophobic interactions with the active site of CYP51 compared to the other ligands, potentially contributing to its robust binding affinity.

**Figure 13:**
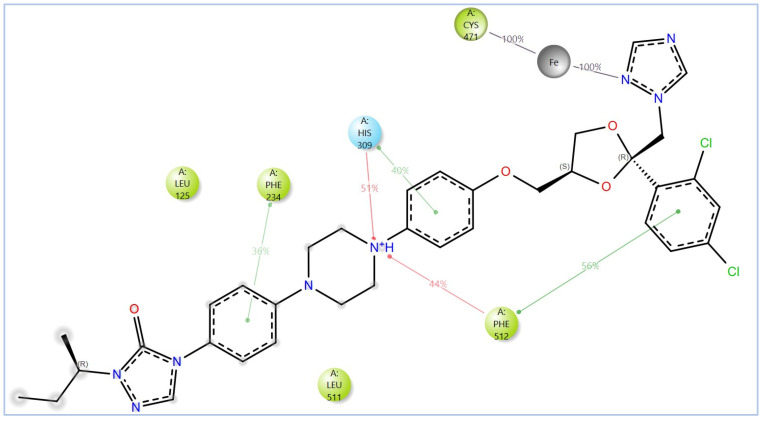
The 2D interactions of itraconazole with the in-house homology model of *Madurella mycetomatis* CYP51. Only interactions occurring for more than 30% of the simulation time in the selected trajectory (0 to 100 ns) are shown.

**Figure 14:**
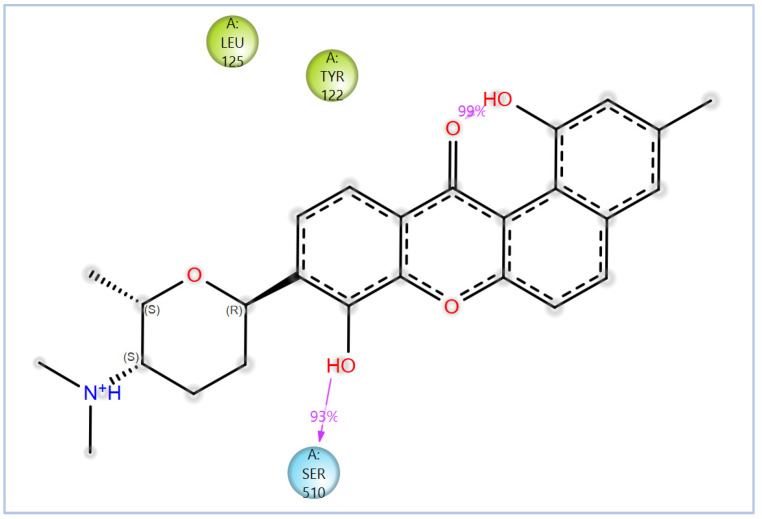
The 2D interactions of NPA029352 with the in-house homology model of *Madurella mycetomatis* CYP51. Only interactions occurring for more than 30% of the simulation time in the selected trajectory (0 to 100 ns) are shown.

**Figure 15:**
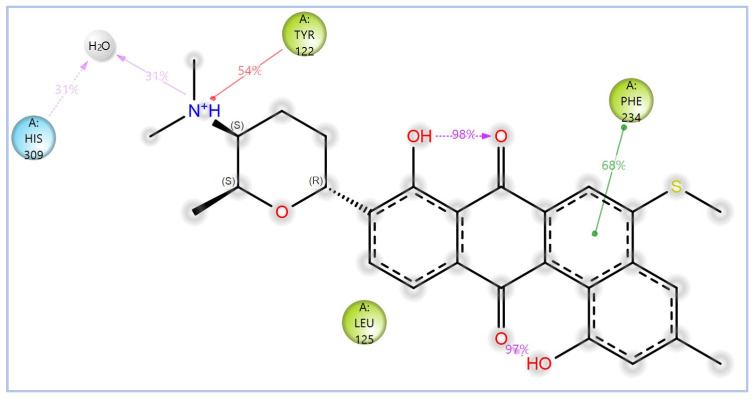
The 2D interactions of NPA029353 with the in-house homology model of *Madurella mycetomatis* CYP51. Only interactions occurring for more than 30% of the simulation time in the selected trajectory (0 to 100 ns) are shown.

**Figure 16:**
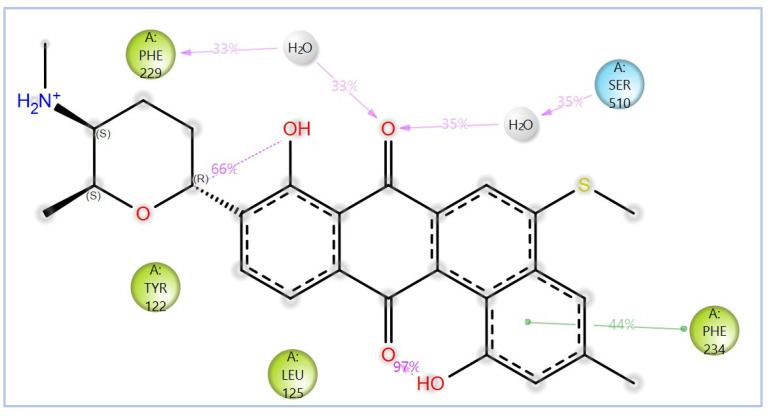
The 2D interactions of NPA029354 with the in-house homology model of *Madurella mycetomatis* CYP51. Only interactions occurring for more than 30% of the simulation time in the selected trajectory (0 to 100 ns) are shown.

The variability in the hydrophobic contact profile among the tested ligands reflects differences in their binding modes and interaction stability within the active site. To this end, the binding interactions of NPA029352, NPA029353, and NPA029354 in complex with the *Madurella mycetomatis* CYP51 homology model were analyzed relative to itraconazole. Stable interactions of itraconazole with the active site were consistently observed throughout the simulation, indicating strong binding affinity. These interactions included pi-cation interactions with His309 (51% of the simulation time) and Phe512 (44%), pi-pi stacking with His309 (40%), Phe234 (36%), and Phe512 (56%), and crucial metal coordination with the heme iron via Cys471 (100%), underscoring its strong affinity. Additionally, it exhibited hydrophobic interactions with Leu125 (44%) and Leu511 (46%), further stabilizing its binding (Figure 13). Among the natural products, NPA029352 formed a strong hydrogen bond with Ser510 (93%) and engaged in hydrophobic interactions with Tyr122 (45%) and Leu125 (31%), suggesting moderate binding stability (Figure 14). NPA029353 displayed diverse interactions, including a water bridge hydrogen bond with His309 (31%), pi-cation interaction with Tyr122 (54%), pi-pi stacking with Phe234 (68%), and hydrophobic contact with Leu125 (36%), indicating a stable binding mode (Figure 15). NPA029354 exhibited water bridge hydrogen bonds with Phe229 (33%) and Ser510 (35%), pi-pi stacking with Phe234 (44%), and hydrophobic interactions with Tyr122 (46%) and Leu125 (48%), highlighting a balance of polar and nonpolar interactions (Figure 16).

While all three natural products interacted with key active-site residues, they lacked the metal coordination with heme iron observed for itraconazole, a key factor in potent CYP51 inhibition. Notably, the heme group in the apo enzyme formed a strong hydrogen bond (2.30 Å) with Tyr122, highlighting the residue’s role in active-site stability (Figure 2 and Supplementary Table S1). The binding of the tested natural products to Tyr122 may modulate this stability, influencing CYP51 inhibition. Strengthening the Tyr122-inhibitor interaction could enhance binding affinity and inhibitory effects, while disrupting the Tyr122-heme interaction may alter heme coordination and affect enzyme function. These interactions suggest a key role for Tyr122 in modulating CYP51 inhibition, warranting further investigation to optimize the inhibitory potential of these natural products. In addition to the aforementioned residues, itraconazole and the three natural products interacted with a mix of common and distinct residues in the CYP51 active site (Supplementary Figure S1). These interactions, involving both electrostatic and hydrophobic bonds, were observed for less than 30% of the simulation time. These findings suggest that NPA029352, NPA029353, and NPA029354 exhibit promising binding characteristics and may serve as potential CYP51 inhibitors, though further optimization may be required to enhance heme coordination for improved inhibitory potency [71].

Based on the findings discussed, our study identified three microbial-based natural products as potential *Madurella mycetomatis* CYP51 inhibitors: two anthraquinones, monacyclinone H (NPA029353) and monacyclinone I (NPA029354), and a xanthone, monacyclinone G (NPA029352). Anthraquinones are widely recognized for their antifungal properties, with documented anti-eumycetoma activity, though their exact mechanism remains unclear [101,102,103]. Our findings suggest that their activity may be mediated through CYP51 inhibition, offering new insights into their mode of action. Similarly, xanthones have been reported as antifungal agents, with a proposed mechanism involving the disruption of ergosterol biosynthesis and cell wall integrity [104]. Our study indicates that xanthone derivatives can also function as a CYP51 inhibitor in *Madurella mycetomatis*, further reinforcing their role in ergosterol synthesis inhibition. These findings align with existing literature and highlight both anthraquinones and xanthones as promising candidates for eumycetoma treatment.

### 2.5. Study Limitations and Future Perspectives

This study presents several limitations and outlines important directions for future research. A primary limitation is its reliance on computational methods to predict compound efficacy. While computational studies offer valuable insights, their results do not always translate accurately to the complexity of real biological systems. The homology model of *Madurella mycetomatis* CYP51 used in this study, though validated, is based on computational predictions due to the absence of an experimentally resolved structure. This modeling approach may introduce subtle inaccuracies that could impact the reliability of binding affinity predictions. Therefore, the natural compounds identified through in silico screening must undergo experimental validation to confirm their therapeutic potential. Both in vitro and in vivo testing are essential to assess their efficacy and safety in treating eumycetoma. In this regard, an enzyme inhibition assay can be used to experimentally validate the predicted interactions. This involves cloning, expressing, and purifying the target enzyme (*Madurella mycetomatis* CYP51), and then assessing its activity in the presence of the test compounds identified through docking. Using appropriate substrates—often measured by HPLC, LC-MS, or spectrophotometry—the assay quantifies how effectively each compound inhibits enzyme activity. By determining IC_50_ or Ki values, experimental inhibition potency can be compared with computational binding affinities. A strong correlation supports the docking predictions, while additional methods like thermal shift assays can further confirm compound-enzyme binding. Moreover, in vitro, studies could be used to investigate the potential development of resistance in *Madurella mycetomatis* through directed evolution—exposing the fungus to subinhibitory concentrations of the compounds over multiple generations allows observation of whether adaptive mutations arise that reduce treatment effectiveness. For the most promising candidates, pharmacokinetic studies in animal models should be conducted to evaluate parameters such as bioavailability, half-life, tissue distribution (especially in infected regions), and routes of elimination. Techniques like HPLC or mass spectrometry can be employed to measure compound concentrations in plasma and tissues, helping to determine appropriate dosing regimens. Additionally, acute toxicity testing in animals can provide estimates of the median lethal dose (LD_50_) and help identify any immediate adverse effects after a single dose. Acquiring an experimentally resolved crystal structure of *Madurella mycetomatis* CYP51 would significantly enhance the precision of future computational studies. Moreover, expanding the screening to larger compound libraries and investigating potential synergistic interactions with existing antifungal drugs could boost therapeutic outcomes. Comprehensive pharmacological profiling, including off-target risk assessments, will be crucial for further development. Finally, exploring other molecular targets within *Madurella mycetomatis* may reveal alternative or complementary treatment strategies that could improve the overall management of eumycetoma.

## 3. Material and Methods

The in-house homology model of *Madurella mycetomatis* CYP51 was constructed using the YASARA (Yet Another Scientific Artificial Reality Application) program (http://www.yasara.org/general.htm, accessed on 27 December 2024) [22]. All subsequent computational analyses, including protein and ligand preparation, virtual screening, molecular docking, MM-GBSA calculations, ADMET analysis, and MD simulations, were performed within the Maestro interface of the Schrödinger suite (Schrödinger Release 2023-1: Schrödinger, LLC, New York, NY, USA, 2023).

The homology model of *Madurella mycetomatis* CYP51 was prepared using the Protein Preparation Wizard from Schrödinger [105]. This process involved adding hydrogens, assigning bond orders, forming zero-order bonds to metals, creating disulfide bonds, removing water molecules more than 5 Å from hetero groups, and capping termini. Epik was used to determine the protonation states of heteroatoms, ensuring that hydrogen atoms were assigned as they would occur at a physiological pH of 7.0 ± 2.0 [106]. Hydrogen bonding networks and water orientations were optimized, followed by structural refinement via energy minimization using the OPLS4 force field [107]. The minimization was performed using the steepest descent algorithm for 100 steps, with non-hydrogen atoms constrained to an RMSD of 0.3 Å, which served as the convergence criterion for terminating the process. The heme was retained within the active site throughout this preparation. To identify the binding pocket of the *Madurella mycetomatis* CYP51 homology model, the SiteMap tool (Schrödinger Release 2023-1: SiteMap, Schrödinger, LLC, New York, NY, USA, 2023) was used [62]. The binding site with the highest Dscore (1.307) was selected for receptor grid generation and subsequent docking studies using the Glide program [108]. A receptor grid (10 Å × 10 Å × 10 Å) was generated around the predicted binding site using the Receptor Grid Generation tool in the Maestro suite, with all other parameters set to default. The grid was designed to accommodate ligands up to 20 Å in length, allowing full representation of ligand flexibility while ensuring that key interacting residues within the active site were encompassed.

Microbial-derived natural products from the Atlas (NPAtlas) database [47,48,49] were processed using the LigPrep tool (Schrödinger Release 2023-1: LigPrep, Schrödinger, LLC, New York, NY, USA, 2023). LigPrep generated and energy-minimized 3D conformations, accounting for all possible tautomers and ionization states at pH 7.0 ± 2.0, using the OPLS4 force field. Virtual screening was performed using the Glide program [108], utilizing a flexible ligand docking approach on the prepared compounds’ library according to the Virtual Screening Workflow (VSW) protocol [109]. Docking occurred in three stages: High-Throughput Virtual Screening (HTVS), Standard Precision (SP), and Extra Precision (XP), with default parameters unchanged. During the docking process, the potential for the nonpolar regions of the target was softened by adjusting the scaling factor of the van der Waals radii to 0.80, this scaling is generally used to reduce the penalty for close contacts with a cut-off value of 0.15 with a partial atomic charge of 0.25.

Natural products that advanced to the XP stage were rescored for binding energy using Prime software (Schrödinger Release 2023-1: Prime, Schrödinger, LLC, New York, NY, USA, 2023) [110]. Binding free energy calculations were carried out using the Prime MM-GBSA method, employing the Variable Dielectric Surface Generalized Born (VSGB) solvent model. In this approach, Prime applies a surface-generalized Born (SGB) model that uses a Gaussian surface, rather than the conventional van der Waals surface, to more accurately represent the solvent-accessible surface area and improve the accuracy of solvation energy estimations.

The computational tool QikProp (Schrödinger Release 2023-1: QikProp, Schrödinger, LLC, New York, NY, USA, 2023) was employed to assess the ADMET profiles and drug-likeness descriptors of the top 9 hits identified through VSW and MM-GBSA calculations [84]. Ligand structures were prepared using the LigPrep tool to optimize 3D geometries and assign protonation states at a physiological pH of 7.4. Predictions were generated using the default settings of QikProp, which evaluates key parameters such as LogP, solubility (LogS), CNS permeability, human oral absorption, and toxicological risks. All calculations were performed in standalone mode within the Schrödinger software (Schrödinger Release 2023-1: Schrödinger, LLC, New York, NY, USA, 2023) environment. The top three natural products, along with the reference drug itraconazole, were selected for further MD simulations using the Desmond simulation package from Schrödinger [111].

Molecular dynamics (MD) simulations were performed on the apo form of the homology model of *Madurella mycetomatis* CYP51, as well as its complexes with NPA029352, NPA029354, 54NPA029354, and itraconazole. Each system was solvated using the SPC water model and placed in an orthorhombic water box with dimensions of 10 Å × 10 Å × 10 Å to ensure complete solvation. Counterions (Na^+^ or Cl^−^) were added to neutralize net charges, along with 0.15 M NaCl to mimic physiological conditions. Energy and volume minimization was conducted using the OPLS4 force field, with a convergence threshold of 1.0 kcal/mol/Å and a maximum of 2000 iterations to ensure system stability. Pre-equilibration followed Desmond’s default relaxation protocol. Simulations were run in the isothermal–isobaric (NPT) ensemble for 100 nanoseconds (ns) at 300 K and 1.01325 bar. Temperature and pressure were maintained using the Nose–Hoover chain thermostat and the Martyna–Tobias–Klein barostat, respectively. Trajectory data were recorded every 100 picoseconds (ps), resulting in 1000 frames per simulation.

## 4. Conclusions

In conclusion, this study successfully identified microbial-based natural products as promising inhibitors of *Madurella mycetomatis* CYP51, offering potential therapeutic alternatives for the treatment of eumycetoma. Through a comprehensive virtual screening approach, including SP and XP docking, MM-GBSA calculations, and ADMET analysis, we highlighted NPA029352, NPA029353, and NPA029354 (monacyclinone G, H, and I, respectively) as top candidates with favorable binding profiles and drug-like properties. Furthermore, MD simulations provided additional support for their stability. These findings suggest that these natural products could serve as valuable starting points for the development of new antifungal therapies for eumycetoma, a neglected and challenging infection. However, the study’s limitations include reliance on an in silico model and the lack of experimental validation. Future research should focus on in vitro and in vivo testing, optimizing drug properties, and exploring combination therapies to improve treatment outcomes.

## Figures and Tables

**Table 1:** XP docking scores and MM-GBSA binding free energies of the top-ranked microbial-based natural products along with itraconazole’s (ionized and unionized).

Compound		Chemical Structure	Docking Score (kcal/mol)	MM-GBSA (kcal/mol)
ID	Name			
NPA020764	Octacosamicin A	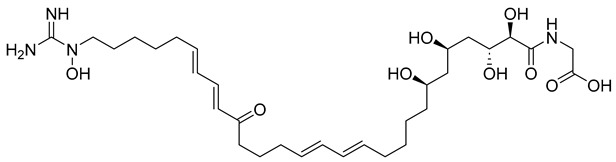	−11.385	−76.66
NPA029353	Monacyclinone H	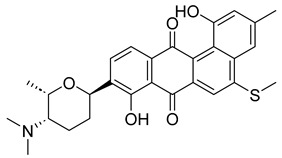	−11.649	−72.39
NPA029354	Monacyclinone I	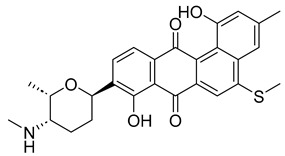	−11.460	−65.62
NPA017021	Monacyclinone E	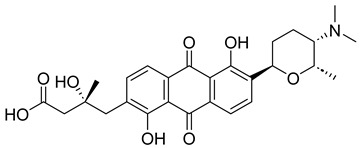	−13.669	−62.35
NPA026108	3′-N-methyl-medermycin	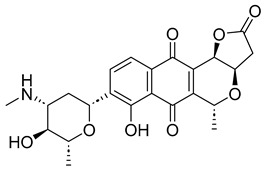	−10.299	−62.10
NPA029352	Monacyclinone G	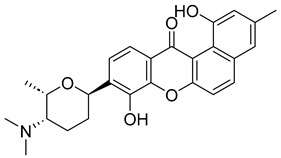	−11.716	−58.89
NPA020514	gamma-iso-Rubromycin	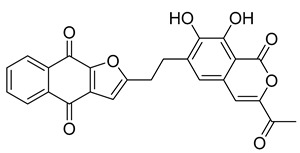	−10.877	−55.05
NPA023185	2,2′-bis-(7-methyl-1,4,5-trihydroxy-anthracene-9,10-dione)	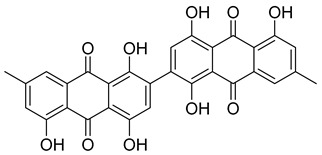	−14.449	−54.51
NPA018887	Roseobacticide K	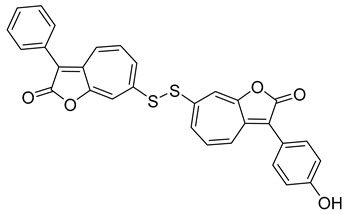	−9.626	−54.34
-	Itraconazole-ionized	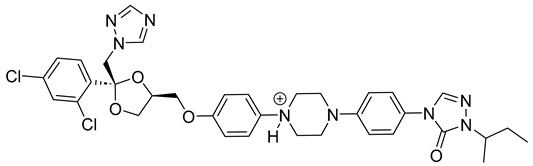	−5.952	−54.07
-	Itraconazole-unionized	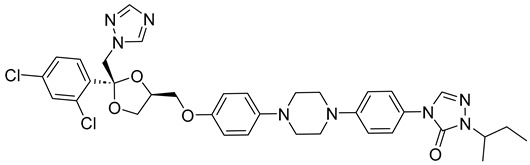	−6.565	−36.07

**Table 2:** Drug-like properties of the top 9 microbial natural products obtained from QikProp.

Molecule	Stars	mol_MW	SASA	donorHB	accptHB	QPlogPo/w	QPlogS	QPlogHERG	QPPCaco	QPlogBB	#metab	QPlogKhsa	% Oral Absorption	PSA	Rule of Five	Rule of Three
NPA017021	0	497.54	769.51	1	8.95	1.10	−5.01	−4.20	2.35	−2.15	8	0.25	40.04	166.75	0	2
NPA018887	1	522.58	691.09	1	6.75	4.89	−6.03	−5.43	594.45	−0.64	1	0.86	92.32	98.02	1	1
NPA020514	0	444.39	732.41	1	9.5	1.71	−5.04	−6.41	17.05	−2.95	5	−0.04	59.01	172.60	0	1
NPA020764	12	624.77	1218.33	8.25	15.75	1.54	−4.94	−4.09	0.03	−8.82	10	−1.14	0	278.04	3	2
NPA023185	4	538.46	799.47	0	6.5	3.26	−6.82	−6.29	2.36	−4.07	8	0.80	26.84	219.93	2	3
NPA026108	2	443.45	701.58	2	13.35	−0.20	−2.70	−5.66	21.16	−1.42	9	−0.56	49.45	161.75	0	2
NPA029352	0	433.50	699.01	2	7.7	3.11	−4.47	−6.18	242.72	−0.26	6	0.50	87.87	83.35	0	0
NPA029353	0	491.60	769.96	1	8.7	3.53	−5.26	−6.15	128.76	−0.59	6	0.62	85.38	98.81	0	0
NPA029354	0	477.57	782.85	2	8.2	3.53	−5.72	−6.55	114.73	−0.71	6	0.68	84.49	105.81	0	1

**Table 3:** A detailed analysis of the PL-RMSD, P-RMSF, and protein-ligand contacts values, including minimum, maximum, and average for the complexes of itraconazole, NPA029352, NPA029353, and NPA029354 with the in-house homology model of *Madurella mycetomatis* CYP51. This also includes comparative data for the apo-protein.

CYP51 Complex	Apo	Itraconazole	NPA029352	NPA029353	NPA029354
PL-RMSD (Å)					
average	3.1	3.2	3.1	3.1	2.7
maximum	4.2	3.7	4.1	3.8	3.5
minimum	1.5	1.5	1.2	1.4	1.3
P-RMSF (Å)					
average	1.2	1.0	1.2	1.2	1.1
maximum	9.7	6.3	8.4	7.4	6.9
minimum	0.5	0.4	0.4	0.4	0.4
H-bond contacts					
average	-	0.5	1.2	0.2	0.4
maximum	-	3.0	3.0	2.0	2.0
minimum	-	0.0	0.0	0.0	0.0
Hydrophobic contacts					
average	-	3.1	2.4	1.7	2.3
maximum	-	8.0	7.0	6.0	7.0
minimum	-	0.0	0.0	0.0	0.0

## Data Availability

The original contributions presented in this study are included in the article/Supplementary Materials. Further inquiries can be directed to the corresponding author.

## Supplementary Materials

The following supporting information can be downloaded at: https://www.mdpi.com/article/10.3390/ph18040598/s1, Table S1: XP molecular docking interactions between the top-ranked microbial-based natural products and itraconazole (ionized and unionized forms) with the in-house homology model of *Madurella mycetomatis* CYP51. The interactions of the heme group are retrieved from the apo form of the model. Figure S1: Interactions of the in-house homology model of *Madurella mycetomatis* CYP51 residues with itraconazole (A), NPA029352 (B), NPA029353 (C) and NPA029354 (D) throughout the entire MD simulations run.

## References

[1] Clark J.E., Kim H.Y., van de Sande W.W.J., McMullan B., Verweij P., Alastruey-Izquierdo A., Chakrabarti A., Harrison T.S., Bongomin F., Hay R.J. (2024). Eumycetoma causative agents: A systematic review to inform the World Health Organization priority list of fungal pathogens. Med. Mycol..

[2] Zijlstra E.E., van de Sande W.W.J., Welsh O., Mahgoub E.S., Goodfellow M., Fahal A.H. (2016). Mycetoma: A unique neglected tropical disease. Lancet Infect. Dis..

[3] Elkheir L.Y.M., Haroun R., Mohamed M.A., Fahal A.H. (2020). Madurella mycetomatis causing eumycetoma medical treatment: The challenges and prospects. PLoS Negl. Trop. Dis..

[4] Fahal A., Mahgoub E.L.S., El Hassan A.M., Abdel-Rahman M.E. (2015). Mycetoma in the Sudan: An update from the Mycetoma Research Centre, University of Khartoum, Sudan. PLoS Negl. Trop. Dis..

[5] Elkheir L.Y.M., Delaye P.-O., Penichon M., Eadie K., Mohamed M.A., Besson P., Chesnay A., Desoubeaux G., Roger S., van de Sande W.W.J. (2024). Emerging therapeutics: The imidazo[1,2-b]pyridazine scaffold as a novel drug candidate for eumycetoma, a neglected tropical disease. Eur. J. Med. Chem..

[6] Suleiman S.H., Wadaella E.L.S., Fahal A.H. (2016). The Surgical Treatment of Mycetoma. PLoS Negl. Trop. Dis..

[7] Agarwal P., Jagati A., Rathod S.P., Kalra K., Patel S., Chaudhari M. (2021). Clinical Features of Mycetoma and the Appropriate Treatment Options. Res. Rep. Trop. Med..

[8] Efared B., Tahiri L., Boubacar M.S., Atsam-Ebang G., Hammas N., Hinde E.F., Chbani L. (2017). Mycetoma in a non-endemic area: A diagnostic challenge. BMC Clin. Pathol..

[9] Emmanuel P., Dumre S.P., John S., Karbwang J., Hirayama K. (2018). Mycetoma: A clinical dilemma in resource limited settings. Ann. Clin. Microbiol. Antimicrob..

[10] Abd Algaffar S.O., Satyal P., Ashmawy N.S., Verbon A., van de Sande W.W.J., Khalid S.A. (2024). In Vitro and In Vivo Wide-Spectrum Dual Antimycetomal Activity of Eight Essential Oils Coupled with Chemical Composition and Metabolomic Profiling. Microbiol. Res..

[11] Chandler D.J., Bonifaz A., van de Sande W.W.J. (2023). An update on the development of novel antifungal agents for eumycetoma. Front. Pharmacol..

[12] Gupta A.K., Lyons D.C. (2015). The Rise and Fall of Oral Ketoconazole. J. Cutan. Med. Surg..

[13] Venugopal P.V., Venugopal T.V. (1993). Treatment of eumycetoma with ketoconazole. Australas. J. Dermatol..

[14] Hussein S.M.E., Saeed A.A., Fahal A.H. (2025). Mycetoma managment: Therapeutic challenges and the role of pharmacovigilance. PLoS Negl. Trop. Dis..

[15] Kwizera R., Bongomin F., Meya D.B., Denning D.W., Fahal A.H., Lukande R. (2020). Mycetoma in Uganda: A neglected tropical disease. PLoS Negl. Trop. Dis..

[16] Emery D., Denning D.W. (2020). The global distribution of actinomycetoma and eumycetoma. PLoS Negl. Trop. Dis..

[17] Siddig E.E., Ahmed A., Ali Y., Bakhiet S.M., Mohamed N.S., Ahmed E.S., Fahal A.H. (2021). Eumycetoma Medical Treatment: Past, Current Practice, Latest Advances and Perspectives. Microbiol. Res..

[18] Abdallh M.G.A., Hemeda S., Elmadani M., Ibrahim B., Ahmed A.E.E. (2025). Epidemiology, risk factors, and awareness of mycetoma among residents in Eastern Sinnar locality, Sudan, 2021. J. Glob. Health.

[19] Hassan R., Cano J., Fronterre C., Bakhiet S., Fahal A., Deribe K., Newport M. (2022). Estimating the burden of mycetoma in Sudan for the period 1991-2018 using a model-based geostatistical approach. PLoS Negl. Trop. Dis..

[20] Siddig E.E., Ahmed A. (2024). The urgent need for developing and implementing a multisectoral One Health strategy for the surveillance, prevention, and control of eumycetoma. IJID One Health.

[21] Hernández-Hernández F., Méndez-Tovar L.J., Frías-De-León M.G., Brunner-Mendoza C., Reyes-Montes M.d.R., Duarte-Escalante E. (2022). Eumycetoma and Global Warming. The Impact of Climate Change on Fungal Diseases.

[22] Mohamed M.A., Awadalla M.K., Mohamed M.S., Elsaman T., Eltayib E.M. (2025). Repurposing FDA-Approved Drugs for Eumycetoma Treatment: Homology Modeling and Computational Screening of CYP51 Inhibitors. Int. J. Mol. Sci..

[23] Zhang R., Wang Y., Wu A., Wang J., Zhang J. (2023). Strategies of targeting CYP51 for IFIs therapy: Emerging prospects, opportunities and challenges. Eur. J. Med. Chem..

[24] Hargrove T.Y., Lamb D.C., Wawrzak Z., Hull M., Kelly S.L., Guengerich F.P., Lepesheva G.I. (2024). Identification of Potent and Selective Inhibitors of Acanthamoeba: Structural Insights into Sterol 14α-Demethylase as a Key Drug Target. J. Med. Chem..

[25] Hossain C.M., Ryan L.K., Gera M., Choudhuri S., Lyle N., Ali K.A., Diamond G. (2022). Antifungals and Drug Resistance. Encyclopedia.

[26] Choi J.Y., Podust L.M., Roush W.R. (2014). Drug Strategies Targeting CYP51 in Neglected Tropical Diseases. Chem. Rev..

[27] Zhang J., Li L., Lv Q., Yan L., Wang Y., Jiang Y. (2019). The Fungal CYP51s: Their Functions, Structures, Related Drug Resistance, and Inhibitors. Front. Microbiol..

[28] Zhou S.F., Zhong W.Z. (2017). Drug Design and Discovery: Principles and Applications. Molecules.

[29] Hughes J.P., Rees S., Kalindjian S.B., Philpott K.L. (2011). Principles of early drug discovery. Br. J. Pharmacol..

[30] Mohamed A.A.E., Mohamed K.A.A., Marwa S.S.O., Magdi A.M., Mahmoud M.E.M., Muzamil M.A.H., Malik S.M., Mohammed A.G. (2016). Evaluation of antileishmanial activity of valproic acid against Leishmania donovani: An integrated in silico and in vitro study. World J. Pharm. Sci..

[31] Bano I., Butt U.D., Mohsan S.A.H., Das S., Thomas S., Das P.P. (2023). Chapter 25—New challenges in drug discovery. Novel Platforms for Drug Delivery Applications.

[32] Goupil L.S., McKerrow J.H. (2014). Introduction: Drug Discovery and Development for Neglected Diseases. Chem. Rev..

[33] Fahal A.H., Ahmed K.O., Saeed A.A., Elkhawad A.O., Bakhiet S.M. (2022). Why the mycetoma patients are still neglected. PLoS Negl. Trop. Dis..

[34] Gilbert I.H. (2013). Drug discovery for neglected diseases: Molecular target-based and phenotypic approaches. J. Med. Chem..

[35] Weng H.-B., Chen H.-X., Wang M.-W. (2018). Innovation in neglected tropical disease drug discovery and development. Infect. Dis. Poverty.

[36] López-López E., Barrientos-Salcedo C., Prieto-Martínez F.D., Medina-Franco J.L. (2020). In silico tools to study molecular targets of neglected diseases: Inhibition of TcSir2rp3, an epigenetic enzyme of Trypanosoma cruzi. Adv. Protein Chem. Struct. Biol..

[37] Yasuo N., Ishida T., Sekijima M. (2021). Computer aided drug discovery review for infectious diseases with case study of anti-Chagas project. Parasitol. Int..

[38] Herrera Acevedo C., Scotti L., Feitosa Alves M., Formiga Melo Diniz M.D., Scotti M.T. (2017). Computer-Aided Drug Design Using Sesquiterpene Lactones as Sources of New Structures with Potential Activity against Infectious Neglected Diseases. Molecules.

[39] Adelusi T.I., Oyedele A.-Q.K., Boyenle I.D., Ogunlana A.T., Adeyemi R.O., Ukachi C.D., Idris M.O., Olaoba O.T., Adedotun I.O., Kolawole O.E. (2022). Molecular modeling in drug discovery. Inform. Med. Unlocked.

[40] Gheidari D., Mehrdad M., karimelahi Z. (2024). Virtual screening, ADMET prediction, molecular docking, and dynamic simulation studies of natural products as BACE1 inhibitors for the management of Alzheimer’s disease. Sci. Rep..

[41] Niazi S.K., Mariam Z. (2023). Computer-Aided Drug Design and Drug Discovery: A Prospective Analysis. Pharmaceuticals.

[42] Ece A. (2023). Computer-aided drug design. BMC Chem..

[43] Mushtaq S., Abbasi B.H., Uzair B., Abbasi R. (2018). Natural products as reservoirs of novel therapeutic agents. EXCLI J..

[44] Smit S., Derks Martijn F.L., Bervoets S., Fahal A., van Leeuwen W., van Belkum A., van de Sande Wendy W.J. (2016). Genome Sequence of Madurella mycetomatis mm55, Isolated from a Human Mycetoma Case in Sudan. Genome Announc..

[45] Smyth M.S., Martin J.H. (2000). x ray crystallography. Mol. Pathol..

[46] Meng X.Y., Zhang H.X., Mezei M., Cui M. (2011). Molecular docking: A powerful approach for structure-based drug discovery. Curr. Comput. Aided Drug Des..

[47] Poynton E.F., van Santen J.A., Pin M., Contreras M.M., McMann E., Parra J., Showalter B., Zaroubi L., Duncan K.R., Linington R.G. (2024). The Natural Products Atlas 3.0: Extending the database of microbially derived natural products. Nucleic Acids Res..

[48] van Santen J.A., Poynton E.F., Iskakova D., McMann E., Alsup T.A., Clark T.N., Fergusson C.H., Fewer D.P., Hughes A.H., McCadden C.A. (2021). The Natural Products Atlas 2.0: A database of microbially-derived natural products. Nucleic Acids Res..

[49] van Santen J.A., Jacob G., Singh A.L., Aniebok V., Balunas M.J., Bunsko D., Neto F.C., Castaño-Espriu L., Chang C., Clark T.N. (2019). The Natural Products Atlas: An Open Access Knowledge Base for Microbial Natural Products Discovery. ACS Cent. Sci..

[50] Elsaman T., Mohamed M.A. (2025). Examining Prenylated Xanthones as Potential Inhibitors Against Ketohexokinase C Isoform for the Treatment of Fructose-Driven Metabolic Disorders: An Integrated Computational Approach. Pharmaceuticals.

[51] Elsaman T., Mohamed M.A., Mohamed M.S., Eltayib E.M., Abdalla A.E. (2025). Microbial-based natural products as potential inhibitors targeting DNA gyrase B of Mycobacterium tuberculosis: An in silico study. Front. Chem..

[52] Fahal A.H., Ahmed E.S., Bakhiet S.M., Bakhiet O.E., Fahal L.A., Mohamed A.A., Mohamedelamin E.S.W., Bahar M.E.N., Attalla H.Y., Siddig E.E. (2024). Two dose levels of once-weekly fosravuconazole versus daily itraconazole in combination with surgery in patients with eumycetoma in Sudan: A randomised, double-blind, phase 2, proof-of-concept superiority trial. Lancet Infect. Dis..

[53] Adelusi T.I., Bolaji O.Q., Ojo T.O., Adegun I.P., Adebodun S. (2023). Molecular Mechanics with Generalized Born Surface Area (MMGBSA) Calculations and Docking Studies Unravel some Antimalarial Compounds Using Heme O Synthase as Therapeutic Target. ChemistrySelect.

[54] Mohamed M.A., Elsaman T., Mohamed M.S., Eltayib E.M. (2024). Computational investigations of flavonoids as ALDH isoform inhibitors for treatment of cancer. SAR QSAR Environ. Res..

[55] Mohamed M.A., Elsaman T., Elderdery A.Y., Alsrhani A., Ghanem H.B., Alruwaili M.M., Hamza S.M.A., Mekki S.E.I., Alotaibi H.A., Mills J. (2024). Unveiling the Anticancer Potential: Computational Exploration of Nitrogenated Derivatives of (+)-Pancratistatin as Topoisomerase I Inhibitors. Int. J. Mol. Sci..

[56] van de Waterbeemd H., Gifford E. (2003). ADMET in silico modelling: Towards prediction paradise?. Nat. Rev. Drug Discov..

[57] Mohamed M.A., Alanazi A.F., Alanazi W.A., Elsaman T., Mohamed M.S., Eltayib E.M. (2025). Repurposing of eluxadoline as a SARS-CoV-2 main protease inhibitor: E-Pharmacophore based virtual screening, molecular docking, MM-GBSA calculations, and molecular dynamics simulations studies. J. Appl. Pharm. Sci..

[58] Hollingsworth S.A., Dror R.O. (2018). Molecular Dynamics Simulation for All. Neuron.

[59] Durrant J.D., McCammon J.A. (2011). Molecular dynamics simulations and drug discovery. BMC Biol..

[60] Friggeri L., Hargrove T.Y., Wawrzak Z., Blobaum A.L., Rachakonda G., Lindsley C.W., Villalta F., Nes W.D., Botta M., Guengerich F.P. (2018). Sterol 14α-Demethylase Structure-Based Design of VNI ((R)- N-(1-(2,4-Dichlorophenyl)-2-(1 H-imidazol-1-yl)ethyl)-4-(5-phenyl-1,3,4-oxadiazol-2-yl)benzamide)) Derivatives To Target Fungal Infections: Synthesis, Biological Evaluation, and Crystallographic Analysis. J. Med. Chem..

[61] Krieger E., Vriend G., Spronk C. (2013). YASARA–yet another scientific artificial reality application. YASARA Org.

[62] Halgren T.A. (2009). Identifying and characterizing binding sites and assessing druggability. J. Chem. Inf. Model..

[63] Hargrove T.Y., Wawrzak Z., Guengerich F.P., Lepesheva G.I. (2020). A requirement for an active proton delivery network supports a compound I-mediated C–C bond cleavage in CYP51 catalysis. J. Biol. Chem..

[64] Ma J., Eadie K., Schippers M., Fahal A., Laleu B., Verbon A., van de Sande W.W.J. (2024). Novel Compound MMV1804559 from the Global Health Priority Box Exhibits In Vitro and In Vivo Activity against Madurella mycetomatis. Int. J. Mol. Sci..

[65] Scolding P., Fahal A., Yotsu R.R. (2018). Drug therapy for Mycetoma. Cochrane Database Syst. Rev..

[66] Lim W., Verbon A., van de Sande W. (2022). Identifying novel drugs with new modes of action for neglected tropical fungal skin diseases (fungal skinNTDs) using an Open Source Drug discovery approach. Expert. Opin. Drug Discov..

[67] Elsaman T., Ahmad I., Eltayib E.M., Suliman Mohamed M., Yusuf O., Saeed M., Patel H., Mohamed M.A. (2024). Flavonostilbenes natural hybrids from Rhamnoneuron balansae as potential antitumors targeting ALDH1A1: Molecular docking, ADMET, MM-GBSA calculations and molecular dynamics studies. J. Biomol. Struct. Dyn..

[68] Li J., Abel R., Zhu K., Cao Y., Zhao S., Friesner R.A. (2011). The VSGB 2.0 model: A next generation energy model for high resolution protein structure modeling. Proteins.

[69] Sgobba M., Caporuscio F., Anighoro A., Portioli C., Rastelli G. (2012). Application of a post-docking procedure based on MM-PBSA and MM-GBSA on single and multiple protein conformations. Eur. J. Med. Chem..

[70] Genheden S., Ryde U. (2015). The MM/PBSA and MM/GBSA methods to estimate ligand-binding affinities. Expert. Opin. Drug Discov..

[71] Hoffer L., Muller C., Roche P., Morelli X. (2018). Chemistry-driven Hit-to-lead Optimization Guided by Structure-based Approaches. Mol. Inform..

[72] Albanese S.K., Chodera J.D., Volkamer A., Keng S., Abel R., Wang L. (2020). Is Structure-Based Drug Design Ready for Selectivity Optimization?. J. Chem. Inf. Model..

[73] Ferreira L.L.G., Andricopulo A.D. (2019). ADMET modeling approaches in drug discovery. Drug Discov. Today.

[74] Singh S.S. (2006). Preclinical pharmacokinetics: An approach towards safer and efficacious drugs. Curr. Drug Metab..

[75] Wu F., Zhou Y., Li L., Shen X., Chen G., Wang X., Liang X., Tan M., Huang Z. (2020). Computational Approaches in Preclinical Studies on Drug Discovery and Development. Front. Chem..

[76] Ioakimidis L., Thoukydidis L., Mirza A., Naeem S., Reynisson J. (2008). Benchmarking the Reliability of QikProp. Correlation between Experimental and Predicted Values. QSAR Comb. Sci..

[77] Ntie-Kang F., Lifongo L.L., Mbah J.A., Owono Owono L.C., Megnassan E., Mbaze L.M., Judson P.N., Sippl W., Efange S.M. (2013). In silico drug metabolism and pharmacokinetic profiles of natural products from medicinal plants in the Congo basin. In Silico Pharmacol..

[78] Ntie-Kang F., Mbah J.A., Lifongo L.L., Owono Owono L.C., Megnassan E., Meva’a Mbaze L., Judson P.N., Sippl W., Efange S.M. (2013). Assessing the pharmacokinetic profile of the CamMedNP natural products database: An in silico approach. Org. Med. Chem. Lett..

[79] Sun D., Gao W., Hu H., Zhou S. (2022). Why 90% of clinical drug development fails and how to improve it?. Acta Pharm. Sin. B.

[80] Butnarasu C., Garbero O.V., Petrini P., Visai L., Visentin S. (2023). Permeability Assessment of a High-Throughput Mucosal Platform. Pharmaceutics.

[81] Ertl P., Rohde B., Selzer P. (2000). Fast calculation of molecular polar surface area as a sum of fragment-based contributions and its application to the prediction of drug transport properties. J. Med. Chem..

[82] Prasanna S., Doerksen R.J. (2009). Topological polar surface area: A useful descriptor in 2D-QSAR. Curr. Med. Chem..

[83] Asano D., Takakusa H., Nakai D. (2024). Oral Absorption of Middle-to-Large Molecules and Its Improvement, with a Focus on New Modality Drugs. Pharmaceutics.

[84] Divyashri G., Krishna Murthy T.P., Sundareshan S., Kamath P., Murahari M., Saraswathy G.R., Sadanandan B. (2021). In silico approach towards the identification of potential inhibitors from Curcuma amada Roxb against H. pylori: ADMET screening and molecular docking studies. Bioimpacts.

[85] Fu C., Shi H., Chen H., Zhang K., Wang M., Qiu F. (2021). Oral Bioavailability Comparison of Artemisinin, Deoxyartemisinin, and 10-Deoxoartemisinin Based on Computer Simulations and Pharmacokinetics in Rats. ACS Omega.

[86] Wanat K., Żydek G., Hekner A., Brzezińska E. (2021). In Silico Plasma Protein Binding Studies of Selected Group of Drugs Using TLC and HPLC Retention Data. Pharmaceuticals.

[87] Lexa K.W., Dolghih E., Jacobson M.P. (2014). A structure-based model for predicting serum albumin binding. PLoS ONE.

[88] Wang T., Sun J., Zhao Q. (2023). Investigating cardiotoxicity related with hERG channel blockers using molecular fingerprints and graph attention mechanism. Comput. Biol. Med..

[89] Masimirembwa C.M., Bredberg U., Andersson T.B. (2003). Metabolic Stability for Drug Discovery and Development. Clin. Pharmacokinet..

[90] Twycross R., Ross J., Kotlinska-Lemieszek A., Charlesworth S., Mihalyo M., Wilcock A. (2015). Variability in Response to Drugs. J. Pain. Symptom Manag..

[91] Lipinski C.A. (2004). Lead- and drug-like compounds: The rule-of-five revolution. Drug Discov. Today Technol..

[92] Zhang M.-Q., Wilkinson B. (2007). Drug discovery beyond the ‘rule-of-five’. Curr. Opin. Biotechnol..

[93] Lionta E., Spyrou G., Vassilatis D.K., Cournia Z. (2014). Structure-based virtual screening for drug discovery: Principles, applications and recent advances. Curr. Top. Med. Chem..

[94] Kurczab R., Ali W., Łażewska D., Kotańska M., Jastrzębska-Więsek M., Satała G., Więcek M., Lubelska A., Latacz G., Partyka A. (2018). Computer-Aided Studies for Novel Arylhydantoin 1,3,5-Triazine Derivatives as 5-HT6 Serotonin Receptor Ligands with Antidepressive-Like, Anxiolytic and Antiobesity Action In Vivo. Molecules.

[95] Trösken E.R., Adamska M., Arand M., Zarn J.A., Patten C., Völkel W., Lutz W.K. (2006). Comparison of lanosterol-14α-demethylase (CYP51) of human and Candida albicans for inhibition by different antifungal azoles. Toxicology.

[96] Jäger M.C., Joos F.L., Winter D.V., Odermatt A. (2023). Characterization of the interferences of systemic azole antifungal drugs with adrenal steroid biosynthesis using H295R cells and enzyme activity assays. Curr. Res. Toxicol..

[97] Borjian Boroujeni M., Shahbazi Dastjerdeh M., Shokrgozar M., Rahimi H., Omidinia E. (2021). Computational driven molecular dynamics simulation of keratinocyte growth factor behavior at different pH conditions. Inform. Med. Unlocked.

[98] Oyewusi H.A., Wahab R.A., Akinyede K.A., Albadrani G.M., Al-Ghadi M.Q., Abdel-Daim M.M., Ajiboye B.O., Huyop F. (2024). Bioinformatics analysis and molecular dynamics simulations of azoreductases (AzrBmH2) from Bacillus megaterium H2 for the decolorization of commercial dyes. Environ. Sci. Eur..

[99] Patil R., Das S., Stanley A., Yadav L., Sudhakar A., Varma A.K. (2010). Optimized hydrophobic interactions and hydrogen bonding at the target-ligand interface leads the pathways of drug-designing. PLoS ONE.

[100] Niu X., Lin L., Liu L., Yu Y., Wang H. (2022). Antifungal activity and molecular mechanisms of mulberrin derivatives against Colletotrichum gloeosporioides for mango storage. Int. J. Food Microbiol..

[101] Agarwal S.K., Singh S.S., Verma S., Kumar S. (2000). Antifungal activity of anthraquinone derivatives from Rheum emodi. J. Ethnopharmacol..

[102] Masi M., Evidente A. (2020). Fungal Bioactive Anthraquinones and Analogues. Toxins.

[103] Ma J., Todd M., van de Sande W.W.J., Biersack B. (2023). Antifungal Activity of Natural Naphthoquinones and Anthraquinones against Madurella mycetomatis. Chem. Biodivers..

[104] Pinto E., Afonso C., Duarte S., Vale-Silva L., Costa E., Sousa E., Pinto M. (2011). Antifungal activity of xanthones: Evaluation of their effect on ergosterol biosynthesis by high-performance liquid chromatography. Chem. Biol. Drug Des..

[105] Madhavi Sastry G., Adzhigirey M., Day T., Annabhimoju R., Sherman W. (2013). Protein and ligand preparation: Parameters, protocols, and influence on virtual screening enrichments. J. Comput.-Aided Mol. Des..

[106] Johnston R.C., Yao K., Kaplan Z., Chelliah M., Leswing K., Seekins S., Watts S., Calkins D., Chief Elk J., Jerome S.V. (2023). Epik: pKa and Protonation State Prediction through Machine Learning. J. Chem. Theory Comput..

[107] Lu C., Wu C., Ghoreishi D., Chen W., Wang L., Damm W., Ross G.A., Dahlgren M.K., Russell E., Von Bargen C.D. (2021). OPLS4: Improving Force Field Accuracy on Challenging Regimes of Chemical Space. J. Chem. Theory Comput..

[108] Friesner R.A., Murphy R.B., Repasky M.P., Frye L.L., Greenwood J.R., Halgren T.A., Sanschagrin P.C., Mainz D.T. (2006). Extra Precision Glide:  Docking and Scoring Incorporating a Model of Hydrophobic Enclosure for Protein−Ligand Complexes. J. Med. Chem..

[109] Pandya V., Rao P., Prajapati J., Rawal R.M., Goswami D. (2024). Pinpointing top inhibitors for GSK3β from pool of indirubin derivatives using rigorous computational workflow and their validation using molecular dynamics (MD) simulations. Sci. Rep..

[110] Klyshko E., Kim J.S.-H., McGough L., Valeeva V., Lee E., Ranganathan R., Rauscher S. (2024). Functional protein dynamics in a crystal. Nat. Commun..

[111] Ivánczi M., Balogh B., Kis L., Mándity I. (2023). Molecular Dynamics Simulations of Drug-Conjugated Cell-Penetrating Peptides. Pharmaceuticals.

