# Supplementary material for: Identification of Microbial-Based Natural Products as Potential CYP51 Inhibitors for Eumycetoma Treatment: Insights from Molecular Docking, MM-GBSA Calculations, ADMET Analysis, and Molecular Dynamics Simulations
Source: Pharmaceuticals (Basel). 2025 Apr 20;18(4):598. doi: 10.3390/ph18040598 (PMC12030664; doi:10.3390/ph18040598)
Supplement: Supplementary file 1 [file pharmaceuticals-18-00598-s001.zip › pharmaceuticals-3569193-supplementary.pdf]

Identification of Microbial-based Natural Products as Potential CYP51 Inhibitors for Eumycetoma Treatment: Insights from Molecular Docking, MM-GBSA Calculations, ADMET Analysis, and Molecular Dynamics Simulations

Tilal Elsaman,[1†*] Mohamed Khalid Alhaj Awadalla,[2†] Malik Suliman Mohamed,[3] Eyman Mohamed Eltayib[3] Magdi Awadalla Mohamed,[1*]

[1]*Department of Pharmaceutical Chemistry, College of Pharmacy, Jouf University, Sakaka 72388, Saudi Arabia*

[2]*Pharmacy Program, Wad Medani College of Medical Sciences and Technology, Wad Medani 21111, Gezira, Sudan*

[3]*Department of Pharmaceutics, College of Pharmacy, Jouf University, Sakaka 72388, Saudi Arabia*

[†]*These authors contributed equally to this work*

[*]*To whom correspondence should be addressed: TE (telbashir@ju.edu.sa) and MAM (maelhussein@ju.edu.sa)*

Table S1: XP molecular docking interactions between the top-ranked microbial-based natural products and itraconazole (ionized and unionized forms) with the in-house homology model of *Madurella mycetomatis* CYP51. The interactions of the heme group are retrieved from the apo form of the model.

Compound	Interactions
NPA020764<br>(Octacosamicin A)	H-Bond:<br>Gly69 (2.16 Å), Tyr136 (1.88 Å), Asp507 (2.47 Å) and HEM530 (2.43 Å)<br>Salt bridge:<br>Lys235 (3.31 Å) and HEM530 (4.8 Å)<br>Pi-cation:<br>HEM530 (4.42 Å)<br>Hydrophobic:<br>Ile66, Tyr68, Met70, Leu91, Leu92, Ile121, Tyr122, Leu125, Tyr136, Phe229, Ile232, Pro231, Phe234, Ile372, Met376, Tyr508, Ala509, Leu511 and Phe512
NPA029353<br>(Monacyclinone H)	Pi-cation:<br>Tyr122 (5.89 Å) and HEM530 (5.86 Å and 6.43 Å)<br>Pi-Pi stacking:<br>Phe234 (4.38 Å)<br>Hydrophobic:<br>Tyr68, Leu91, Leu92, Ile121, Tyr122, Va124, Leu125, Phe130, Val135, Tyr136, Phe229, Phe234, Met305, Ala306, Ile372, Met376, Val397, Leu511 and Phe512
NPA029354<br>(Monacyclinone I)	Pi-cation:<br>HEM530 (6.31 Å and 6.56 Å)<br>Hydrophobic:<br>Tyr68, Leu91, Leu92, Ile121, Tyr122, Va124, Leu125, Phe130, Tyr136, Phe229, Phe234, Met305, Ala306, Ile372, Met376, Val397, Leu511 and Phe512
NPA017021<br>(Monacyclinone E)	H-Bond:<br>Leu91 (2.11 Å) and Lys94 (2.24 Å)<br>Salt bridge:<br>Lys94 (3.42 Å)<br>Pi-cation:<br>Tyr122 (5.74 Å) and HEM530 (6.0 Å and 6.47 Å)<br>Hydrophobic:<br>Leu91, Leu92, Ile121, Tyr122, Va124, Leu125, Phe130, Val135, Tyr136, Phe229, Met305, Ala306, Ile372, Met376, Val397, Phe234, Leu511 and Phe512
NPA026108<br>(3'-N-methyl-medermycin)	H-Bond:<br>Lys94 (2.48 Å)<br>Pi-cation:<br>HEM530 (6.23 Å)<br>Hydrophobic:<br>Leu91, Leu92, Ile121, Tyr122, Va124, Leu125, Phe130, Tyr136, Phe229, Phe234, Met305, Ile372, Ile375, Met376, Val397, Leu511 and Phe512
NPA029352<br>(Monacyclinone G)	H-Bond:<br>Ser510 (2.17 Å)<br>Pi-cation:<br>HEM530 (4.56 Å and 5.27 Å)<br>Pi-Pi stacking:<br>Phe234 (3.66 Å and 4.06 Å)<br>Hydrophobic:<br>Tyr68, Leu91, Leu92, Ile121, Tyr122, Va124, Leu125, Phe130, Tyr136, Phe229, Phe234, Met305, Ala306, Ile372, Met376, Val397, Leu511 and Phe512
NPA020514<br>(gamma-iso-Rubromycin)	H-Bond:<br>Ile121 (1.76 Å)<br>Pi-Pi stacking:

	HEM530 (4.69 Å and 5.36 Å)<br>Hydrophobic:<br>Tyr68, Leu91, Leu92, Ile121, Tyr122, Va124, Leu125, Phe130, Val135, Tyr136, Phe229, Phe234, Ala306, Ile372, Met376 and Leu511
NPA023185 (2,2'-bis-(7-methyl-1,4,5-trihydroxy-anthracene-9,10-dione))	Pi-Pi stacking:<br>HEM530 (5.16 Å)<br>Hydrophobic:<br>Tyr68, Leu91, Leu92, Ile121, Tyr122, Val124, Leu125, Phe130, Val135, Tyr136, Phe229, Phe234, Ala302, Leu303, Ala306, Ile372, Ile375, Met376, Leu511 and Phe512
NPA018887 (Roseobacticide K)	H-Bond:<br>Leu91 (2.14 Å)<br>Pi-Pi stacking:<br>HEM530 (5.43 Å)<br>Hydrophobic:<br>Tyr68, Leu91, Leu92, Ile121, Tyr122, Va124, Leu125, Phe130, Val135, Tyr136, Phe234, Ala302, Met305, Ala306, Ile372, Met376, Val397, Leu511 and Phe512
Itraconazole-ionized	Halogen bond:<br>Ser374 (3.19 Å)<br>Pi-cation:<br>Tyr122 (5.87 Å)<br>Pi-Pi stacking:<br>Phe229 (4.74 Å), Phe234 (4.23 Å) and HEM530 (4.27 Å and 4.76 Å)<br>Hydrophobic:<br>Leu91, Leu92, Ile121, Tyr122, Va124, Leu125, Val129, Phe130, Val135, Tyr136, Phe229, Phe234, Ala302, Met305, Ala306, Ile372, Ile375, Met376, Val397, Leu511 and Phe512
Itraconazole-unionized	Halogen bond:<br>Ser374 (3.21 Å)<br>Pi-Pi stacking:<br>Phe229 (4.87 Å), Phe234 (3.88 Å) and HEM530 (4.28 Å and 4.77 Å)<br>Hydrophobic:<br>Tyr68, Leu91, Leu92, Ile121, Tyr122, Leu125, Phe130, Val135, Tyr136, Phe229, Phe234, Ala302, Met305, Ala306, Ile372, Ile375, Met376, Val397, Leu511 and Phe512
Heme group (in the apo form)	H-bond:<br>Tyr122 (2.30 Å), Tyr136 (1.90 Å), Lys147 (2.25 Å), Arg377 (2.23 Å) and His469 (2.15 Å)<br>Ionic bond:<br>Cys471 (2.52 Å)<br>Hydrophobic:<br>Leu154, Leu205, Leu303, Ala306, Gly307, Ser310, Thr314, Leu366, Pro371, Ile372, Ile375, Pro463, Phe464, Ile472, Gly473, Phe476, Ala477 and Leu481

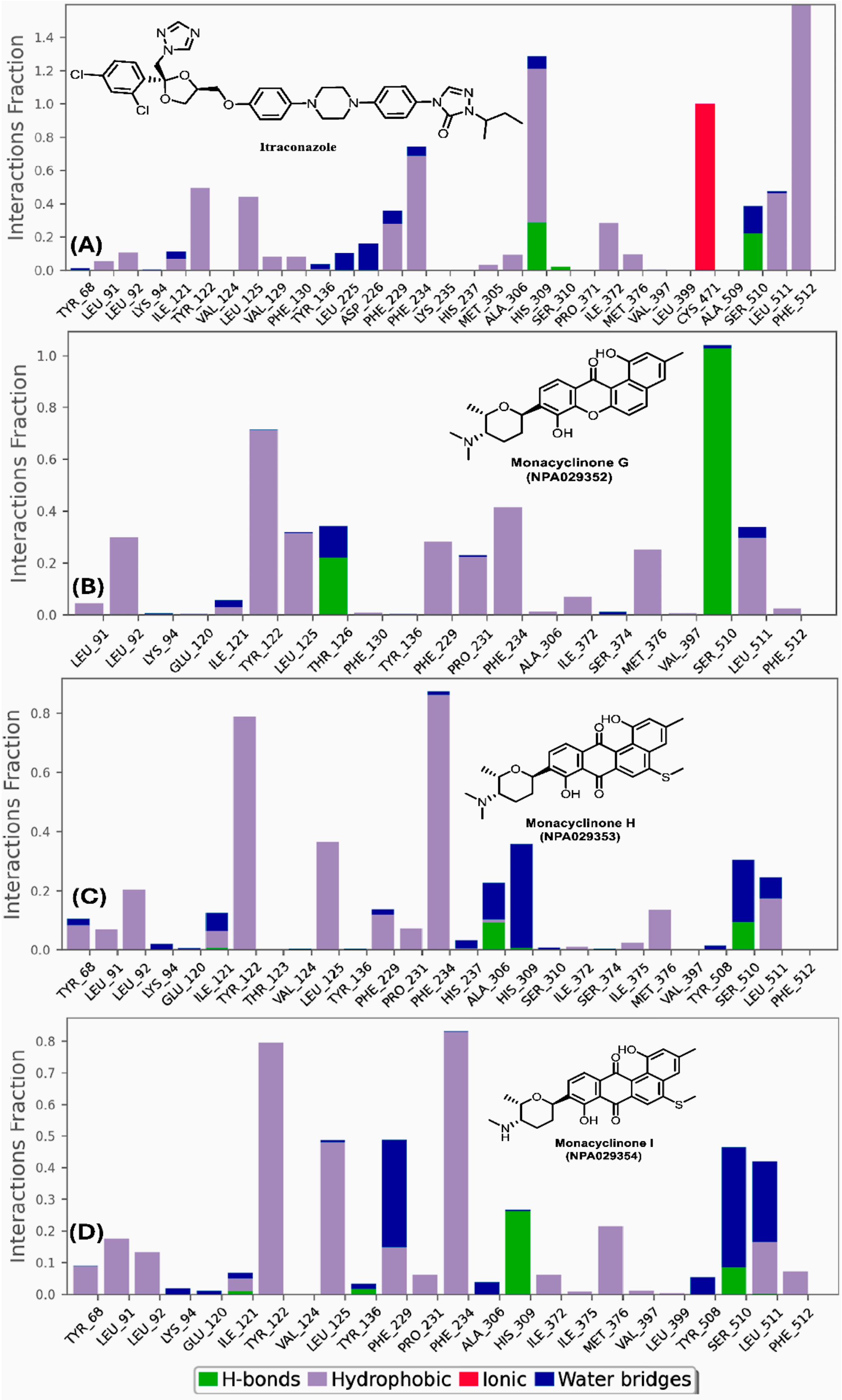

Figure S1: Interactions of the in-house homology model of *Madurella mycetomatis* CYP51 residues with itraconazole (**A**), NPA029352 (**B**), NPA029353 (**C**) and NPA029354 (**D**) throughout the entire MD simulations run.
